# Genome-wide association study identifies favorable SNP alleles and candidate genes for waterlogging tolerance in chrysanthemums

**DOI:** 10.1038/s41438-018-0101-7

**Published:** 2019-02-01

**Authors:** Jiangshuo Su, Fei Zhang, Xinran Chong, Aiping Song, Zhiyong Guan, Weimin Fang, Fadi Chen

**Affiliations:** College of Horticulture, Nanjing Agricultural University, Key Laboratory of Landscape Agriculture, Ministry of Agriculture, 210095 Nanjing, China

**Keywords:** Plant breeding, Plant genetics, Flooding

## Abstract

Chrysanthemums are sensitive to waterlogging stress, and the development of screening methods for tolerant germplasms or genes and the breeding of tolerant new varieties are of great importance in chrysanthemum breeding. To understand the genetic basis of waterlogging tolerance (WT) in chrysanthemums, we performed a genome-wide association study (GWAS) using 92,811 single nucleotide polymorphisms (SNPs) in a panel of 88 chrysanthemum accessions, including 64 spray cut and 24 disbud chrysanthemums. The results showed that the average MFVW (membership function value of waterlogging) of the disbud type (0.65) was significantly higher than that of the spray type (0.55) at *P* < 0.05, and the MFVW of the Asian accessions (0.65) was significantly higher than that of the European accessions (0.48) at *P* *<* 0.01. The GWAS performed using the general linear model (GLM) and mixed linear model (MLM) identified 137 and 14 SNP loci related to WT, respectively, and 11 associations were commonly predicted. By calculating the phenotypic effect values for 11 common SNP loci, six highly favorable SNP alleles that explained 12.85—21.85% of the phenotypic variations were identified. Furthermore, the dosage-pyramiding effects of the favorable alleles and the significant linear correlations between the numbers of highly favorable alleles and phenotypic values were identified (*r*^2^ = 0.45; *P* < 0.01). A major SNP locus (Marker6619-75) was converted into a derived cleaved amplified polymorphic sequence (dCAPS) marker that cosegregated with WT with an average efficiency of 78.9%. Finally, four putative candidate genes in the WT were identified via quantitative real-time PCR (qRT-PCR). The results presented in this study provide insights for further research on WT mechanisms and the application of molecular marker-assisted selection (MAS) in chrysanthemum WT breeding programs.

## Crop genetics: chrysanthemums that cope with water

Researchers in China have identified several genes that help chrysanthemum plants withstand waterlogging. Fadi Chen’s team at Nanjing Agricultural University used high-throughput sequencing to search for correlations between genetic mutations and waterlogging tolerance in 88 chrysanthemum varieties. One analysis method identified 137 mutations linked to waterlogging tolerance, while a second uncovered 14 mutations. Comparing the two lists revealed 11 mutations common to both. Six of these mutations could improve waterlogging response by 25%, and the team found that the mutations could be combined to improve performance even further. The team developed and tested a genetic marker to identify one of the high-tolerance mutations, demonstrating how this information may help breeders. They also identified four genes linked with the 137 mutations. Altogether, these findings point the way towards breeding chrysanthemums with greater waterlogging tolerance.

## Introduction

The chrysanthemum (*Chrysanthemum morifolium* Ramat.) is the second-most important ornamental species in the world, and it accounts for a large proportion of the total commercial production of these plants^1^. Cut chrysanthemums can be divided into the following two types depending on the flower diameter: spray cut type and disbud-cut type. The disbud type bears a large flower of greater than 6 cm and a single flower per stem, and the spray type bears several small flowers of less than 6 cm per stem.

Waterlogging stress is a common constraint in the chrysanthemum industry around the world, particularly in the southern production regions of China^2^. Screening for tolerant germplasms or genes and developing waterlogging-tolerant chrysanthemum cultivars are efficient solutions to this challenge. To genetically improve crops for waterlogging tolerance (WT), the possible mechanisms underlying water stress and the genetic variations associated with WT within a species must be investigated. Previous studies have revealed considerable variations in WT traits in maize^3^, soybeans^4^, barley^5^, and dry beans^6^. Recently, we evaluated the WT of 100 chrysanthemum germplasm resources through two greenhouse experiments and one field experiment, and we screened out 7 accessions that exhibited high WT^7^. However, the traditional screen is gruelling and time-consuming work, and it is also readily affected by environmental factors. Therefore, the breeding of tolerant varieties tends to focus on identifying and exploiting molecular markers that are closely linked to the genes that control the WT trait.

Single nucleotide polymorphisms (SNPs) are defined as single-base changes at a specific nucleotide position, and they are widely distributed throughout genomes in both the coding and noncoding regions of all organisms^8^. SNP markers belong to the third generation of DNA molecular marker technology and have several advantages, including abundance, stability, high-throughput genotyping, and relatively low mutation rates. A large number of SNPs can be identified within a species via high-throughput next generation sequencing (NGS) technologies, such as restriction-site-associated DNA sequencing (RAD-seq)^9^, genotyping-by-sequencing (GBS)^10^, specific-locus amplified fragment sequencing (SLAF-seq)^11^, and double digest RAD-seq^12^. Thus, SNP markers have been widely used in genetic diversity assessments, molecular evolution studies, and genetic mapping for traits of interest in crop species^13^.

In recent years, genome-wide association studies (GWASs) based on linkage disequilibrium have been shown to represent a powerful tool for detecting important QTLs or genes underlying complex traits in the sequenced genomes of rice^14^, *Arabidopsis thaliana*^15^, and maize^16^ and the unsequenced genomes of wheat^17^ and roses^18^. In chrysanthemums, GWASs have been used to identify favorable alleles and to explore the pattern of inheritance for a number of horticultural and WT traits based on gel-based markers^7,19,20^. However, the low marker density exhibited in the traditional molecular marker system limits the detection power of GWASs. Recently, Chong et al.^21^ first attempted to assess the genetic diversity and evolutionary relationships and conducted a GWAS analysis for a set of 199 accessions using > 90,000 SNPs amplified via SLAF-seq, which provides a more efficient method for high-density, sequence-based, and genome-wide polymorphism screening in the chrysanthemum population.

However, SNP assays require expensive equipment or reagents, which has limited the application of SNPs. SNPs can be employed through conversion to other available markers, including single-strand conformation polymorphisms (SSCPs)^22^, high resolution melting (HRM)^23^, allele-specific PCR (ASP)^24^, nucleotide-amplified polymorphisms (SNAPs)^25^, competitive allele-specific PCR (KASP)^26^, and cleaved amplified polymorphic sequences (CAPSs) or derived CAPSs (dCAPSs). Among these options, the CAPS and dCAPS methods have been demonstrated as superior techniques for SNP detection because of their simplicity, efficiency, and economy^25^. The CAPS/dCAPS method, which is also known as the PCR-RFLP marker, is a technique that combines PCR and restriction enzyme digestion to detect restriction fragment length polymorphisms (RFLPs). In detail, CAPSs are based on restriction enzyme site polymorphisms that are detected after the amplification of a locus by PCR. When these restriction sites are not available within the SNP locus, restriction sites can be created during PCR amplification by using primer design to introduce one or more mismatches adjacent to the SNPs of interest, thus creating a synthetic restriction site in the amplified product allele; this method is called dCAPS^27^. For both methods, the PCR products are then subjected to restriction enzyme digestion, and the presence or absence of the SNP is determined by the resulting restriction pattern that is resolved on an agarose or polyacrylamide gel. To date, successfully developed CAPS or dCAPS markers have been linked to different traits in various crops, such as a waxy character in wheat^28^, yellow leaf curl disease resistance in tomatoes^29^, eating quality in rice^25^, head smut resistance in maize^30^, and fiber quality traits in cotton^31^. However, the use of CAPS/dCAPS markers in chrysanthemums has not been reported.

In the present study, a GWAS for the WT of chrysanthemums was performed using an association panel with 88 chrysanthemum accessions, which were genotyped by SLAF-seq^21^. The objectives of this study were to (1) detect SNP loci associated with WT chrysanthemums, (2) mine favorable alleles underlying the WT and assess their pyramiding effects, (3) develop dCAPS markers that cosegregated with WT, and (4) identify potential candidate genes for WT. The results of this study will provide insights into the genetic determination of WT and benefit marker-assisted selection in chrysanthemum breeding.

## Materials and methods

### Plant materials

A total of 88 representative chrysanthemum accessions were used in the present study, including 30 entries developed in Europe, 17 in China, 23 in Japan, 2 in South Korea, and 16 entries of unknown provenance (Supplementary Table S1). Because of the limited number of accessions contained in one geographic origin group, all the accessions developed in China, Japan, and South Korea are categorized as being of Asian origin. In our previous study, the panel was included in a larger collection for the association mapping of certain horticultural characteristics and WT traits based on 707 molecular markers (371 SRAPs, 49 SCoTs, and 287 SSRs)^7,20^. All the materials were maintained at the Chrysanthemum Germplasm Resource Preserving Centre of Nanjing Agricultural University, China.

### Phenotypic evaluation of WT

The WT of the GWAS panel was evaluated using three pot experiments under greenhouse conditions (EXP.1, EXP.2, and EXP.3). The data for EXP.1 and EXP.2 have been previously reported in Su et al.^7^. Another pot experiment (EXP.3) was performed to reduce the environmental factor influence in this study. The experimental design and WT evaluation were described in our earlier report^7^. In brief, the pot experiments followed a randomized complete block design with three replications for the waterlogging treatment and two with normal water management. Three seedlings per entry were included in each replicate. The waterlogging treatments were conducted at the 10–12-leaf stage, and each pot was filled with 3 cm of water above the soil surface. After 3 d of waterlogging stress, the wilting index (WI) was recorded based on a symptom severity scale from 1 to 5, with 1 indicating no symptoms. After 10 d of treatment, the ratio of the dead leaf area (DLR) to the chlorosis score (Score) was recorded. A membership function value of waterlogging (MFVW), which integrated these three measurements, was calculated for each entry and was employed for the subsequent GWAS analysis. The range of the MFVWs was from 0 to 1.0, and a higher MFVW was indicative of enhanced WT. For additional details on the WI, DLR, Score, and MFVW calculation criteria, please refer to Su et al.^7^. Significant correlations (*P* < 0.01) with *r* values ranging from 0.61 to 0.74 and no significant differences (*P* > 0.05) were observed among the three pot experiments (Supplementary Figure S1), and the mean values of the three experiments were used for the subsequent analysis.

### SNP genotyping and population structure evaluation

The SNP genotyping of the association panel was performed using the SLAF-seq approach reported by Chong et al.^21^. The SNPs were filtered using a minor allele frequency (MAF) > 0.05 and an integrity of each SNP > 0.5. As a result, 92,811 SNPs were identified from 468,521 SNPs and were used for further analysis.

A phylogenetic tree of the chrysanthemum accessions based on the informative SNPs was constructed using the neighbor-joining algorithm^32^ in MEGA5^33^. The population structure was assessed using the ADMIXTURE software package according to the minimum cross-validation error value^34^. The kinship matrix was calculated with SPAGeDi software^35^. A principal component analysis (PCA) was performed using Cluster software^36^.

### GWAS and favorable allele identification

Based on the filtered SNP data, the GWAS analysis was conducted using TASSEL v4.0 software^37^ with two models, the general linear model (GLM) considering Q and the mixed linear model (MLM) considering Q and K. The mean values of the three pot experiments were used for the GWAS. The significance threshold for a trait marker association was set at *P* ≤ 0.001. Moreover, the *P* values were also adjusted using the Bonferroni threshold (*P* ≤ 1/92, 811 = 1.08E-5) to reduce false positive associations^38^. The identified SNP loci were named with the SLAF tag followed by a hyphen (-) and the position (e.g., Marker6619-75). The proportion of the phenotypic variation explained (PVE) by each marker was estimated by the relevant *R*^2^. Because a higher MFVW is indicative of an enhanced WT, a phenotypic effect (*a*_i_) > 0 indicates a favorable allele. The *a*_i_ of the SNP loci that was simultaneously detected under two models was estimated by comparing the average phenotypic value of panel accessions harboring favorable alleles with that of accessions harboring unfavorable alleles.

### Development and verification of dCAPS markers linked to the WT

Thirteen waterlogging-tolerant cultivars and thirteen waterlogging-sensitive cultivars were used to convert the SNPs into dCAPS markers and to identify the WT mechanisms. Additionally, 26 F_1_ lines showing an extreme WT derived from‘Nannong Xuefeng’ and ‘Monalisa’ were used to verify the dCAPS markers (Supplementary Table S2)^39^. The genomic DNA of the above materials was extracted from young leaves using the CTAB method^40^. The DNA was diluted to a final concentration of 300 ng/µL and stored at −20 °C until required. The corresponding restriction enzymes were identified using the online enzyme-cutting recognition software dCAPS Finder 2.0^27^, and the specific primers were designed using Primer Premier 5.0 software (PREMIER Biosoft International, CA, US).

The PCR reactions were conducted in a total volume of 25 µL, including 2.5 µL of 10 × Buffer (Mg^2+^ plus), 2.0 µL of dNTP (2.5 mM each), 1 µL of each primer, 1 U of *Taq* DNA polymerase, 1 µL of template DNA, and 17.3 µL of ddH_2_O. The PCR protocol consisted of an initial denaturation at 94 °C/3 min followed by 35 cycles of 94 °C/30 s, 57 °C/30 s, and 72 °C/30 s and finally an elongation step of 72 °C/7 min. The amplified PCR products were sequenced and digested at 37 °C for 3 h in a final volume of 50 µL, including 5 µL of 10 × NEBuffer, 10 µL of the PCR product, 34 µL of ddH_2_O, and 1 µL of restriction endonuclease that was then heat-deactivated according to the manufacturer’s instructions (New England Biolabs, NEB, USA). The digestion products were separated via 10% native polyacrylamide gel electrophoresis and visualized by silver staining.

### Candidate gene annotation and verification

All the SLAF sequences that harbored one or more of the significant SNPs associated with WT were aligned with the available chrysanthemum transcriptome databases using the BlastX algorithm. The potential WT candidate genes were preliminarily identified according to the gene annotation. To verify whether the candidate genes pertained to WT, the selected genes were validated using quantitative real-time PCR (qRT-PCR). Root samples from the waterlogging-tolerant cultivar ‘Nannong Xuefeng’ and the waterlogging-sensitive cultivar ‘Monalisa’ were collected after 12 h of waterlogging stress treatment and normal watering treatment, with three plants in each group. The total RNA was extracted using a Plant RNA Extraction Kit (Aidlab Bio, Beijing, China) according to the manufacturer’s protocol. The primers used for qRT-PCR amplification are listed in Supplementary Table S3, and they were designed to avoid the conserved regions. The qRT-PCR assays were conducted with three biological replicates and three independent technical replicates for each sample using a SYBR Premix Ex Taq™ Kit according to^41^. To normalize the variance among samples, the *EF1α* gene was used as an endogenous control, and the gene expression level was calculated using the 2^−ΔΔCT^ method^42^.

## Results

### Population stratification

**Fig. 1:**
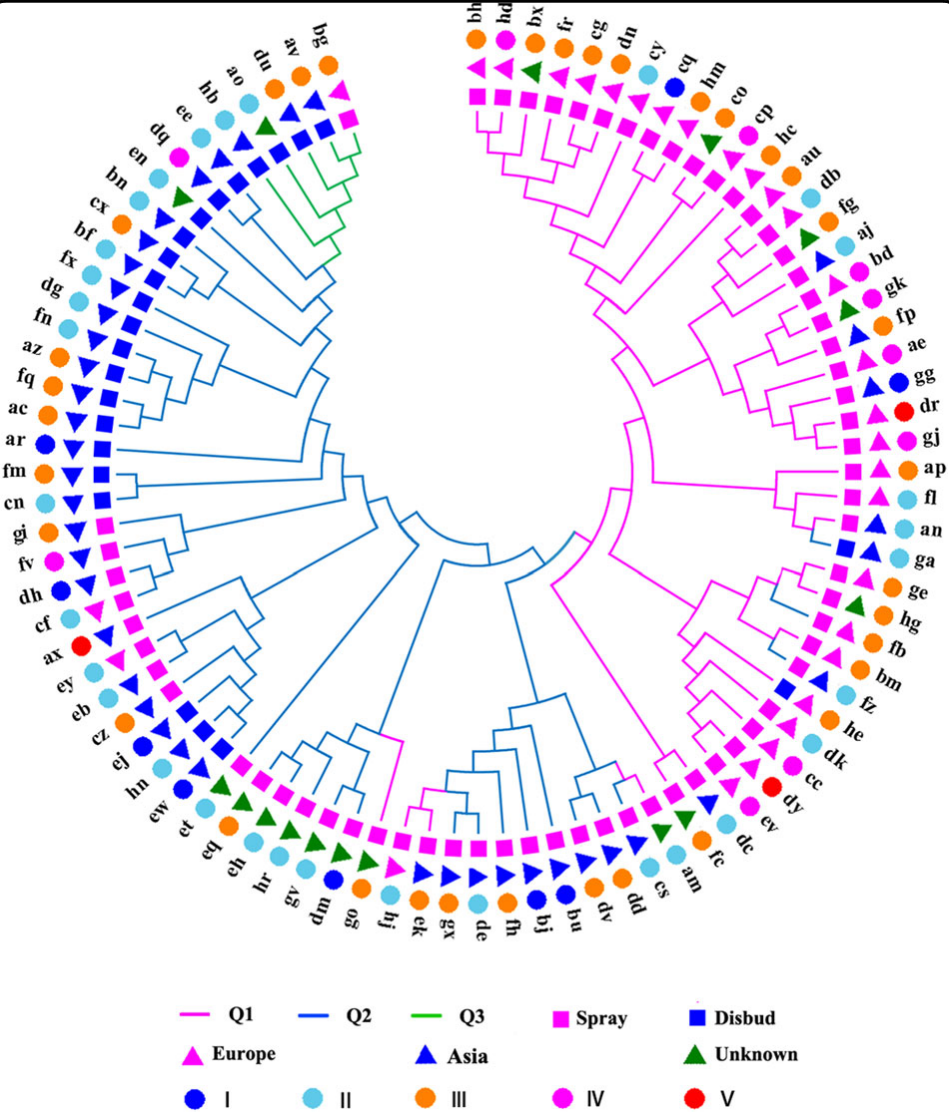
Neighbor-joining phylogenetic tree of the 88-entry germplasm panel constructed using 92,811 SNPs

The genotype data of 92,811 high-quality SNPs were used for the population stratification. The testing accessions could be largely separated into three subpopulations according to the minimum cross-validation error value (Supplementary Figure S2a, b), and the PCA results were essentially consistent with the population structure findings (Supplementary Figure S2c). The phylogenetic relationships between the 88 entries under consideration as the subpopulation, cultivated types, geographic origins, and WT grades are illustrated in Fig. 1. Clearly, the phylogenetic tree assessed the panel into two branches, with one branch including all the entries from subpopulation 1 (Q1) and the other branch including all the entries for subpopulation 2 (Q2) and subpopulation 3 (Q3), both of which had a few exceptions. The spray and disbud-cut chrysanthemum cultivars in the panel could also be clearly separated according to the cultivated type via phylogenetic analysis, i.e., 39 (97.5%) spray cut chrysanthemum accessions were included in Q1 and 79.2% of the disbud cut chrysanthemum accessions were included in Q2. Most of these chrysanthemum accessions from each subpopulation had mixed provenances, with 26 (81.3%) entries of known origin included in Q1 originating from Europe and 33 (91.7%) entries of known origin included in Q2 originating from Asia (Supplementary Figure S3). The results indicated that these accessions might have experienced introgression or gene flow during breeding.

### WT performance

The phenotypic data on the WT across the three experiments for the panel are summarized in Supplementary Figure S1 and Supplementary Table S1. Obvious clusters were not observed based on five WT grades (Fig. 1). However, significant differences in the MFVWs were observed among the subpopulations, cultivated types, and origins (Table 1). Specifically, the average MFVW values of the entries included in Q1, Q2, and Q3 were 0.49, 0.66, and 0.57, respectively. The minimum MFVW was 0.09 in ‘Puma Sunny’, which was included in Q1, whereas the maximum MFVW was 0.89 in ‘Xiwang Zhiguang’, ‘Xiaoli’, ‘Huoyan’, ‘Winter White’, and ‘Qx097’, which were all included in Q2. The MFVW of the disbud-cut chrysanthemum accessions (0.65) was significantly higher than that of the spray cut chrysanthemum accessions (0.55; *P* < 0.05), and the MFVW of the Asia chrysanthemum accessions (0.65) was significantly higher than that of the European chrysanthemum accessions (0.48; *P* < 0.01).

**Table 1:** Average MFVW values according to different classifications

	Subpopulation^a^			Type^a^		Origin^a^	
	Q1	Q2	Q3	Spray	Disbud	Europe	Asia
Mean	0.49Ab	0.66Aa	0.57Aab	0.55Ab	0.65Aa	0.48Bb	0.65Aa
*SD*	0.18	0.15	0.08	0.19	0.12	0.18	0.13
No.	40	43	5	64	24	30	42

^a^Different uppercase letters represent significance at *P* < 0.01, and different lowercase letters represent significance at *P* < 0.05

### GWAS and the mining of highly favorable SNP alleles

**Fig. 2:**
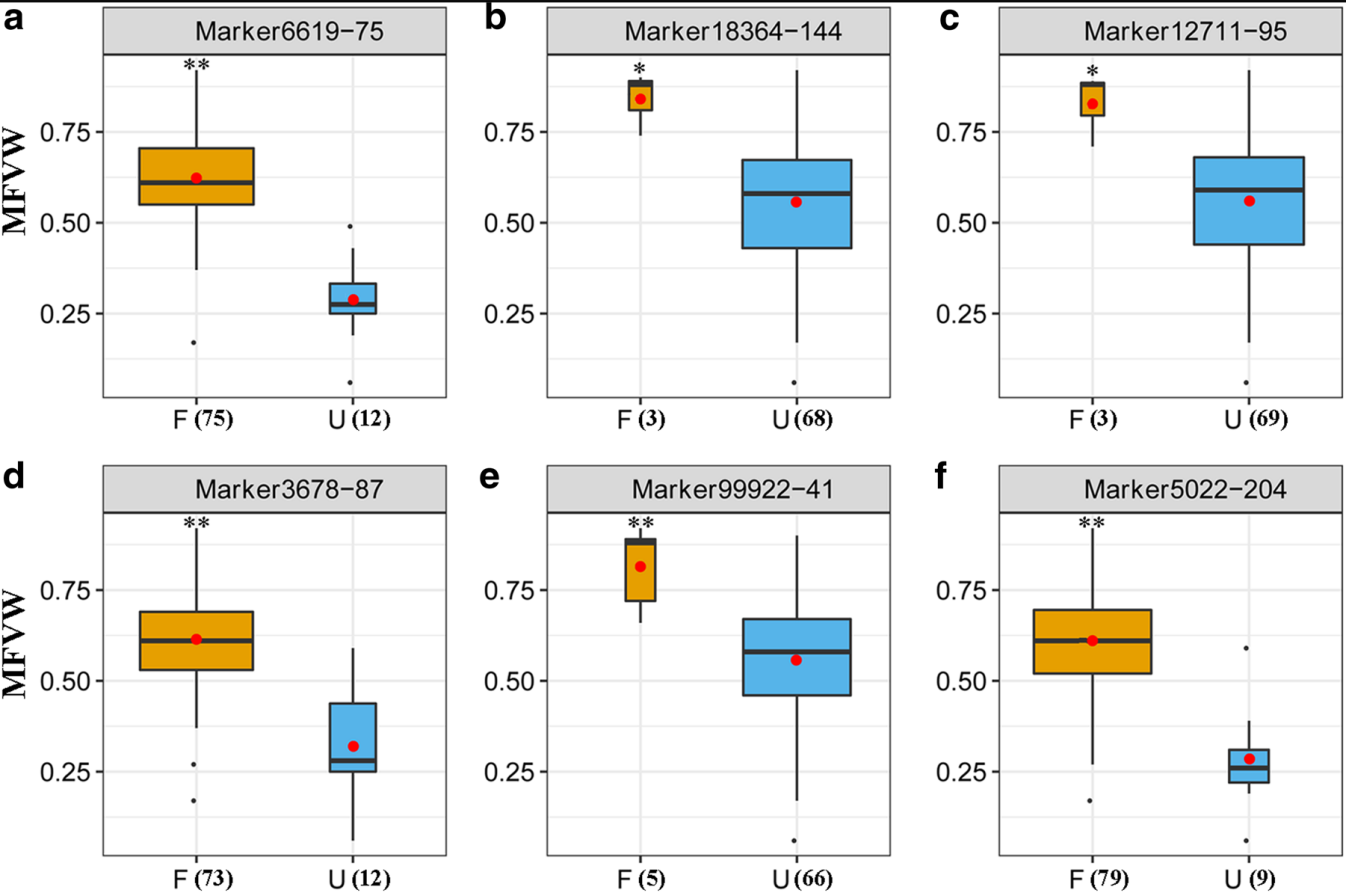
**Box plots indicating the variation in MFVWs for six favorable alleles. a–f**, Boxes colored orange and blue refer to entries carrying the favorable (F) and unfavorable (U) alleles, respectively. The number of individuals for each allele is given in parenthesis and is represented by the width of the box. The mean values of each group are indicated by red circles. ** and * differ at *P* < 0.01 and < 0.05, respectively, as calculated by Student’s *t* test

In total, 137 SNPs were identified as being significantly associated with the WT based on the GLM at a threshold of *P* ≤ 1E-3, and they explained 9.39–21.76% of the phenotypic variance; 14 associations were detected based on the MLM at the same threshold value, and they explained 12.68–23.60% of the phenotypic variance. Among these associations, 11 SNPs were found in both models (Table 2). All the SNPs associated with the WT are summarized in Supplementary Table S4. The *P*-value of a specific association detected via the GLM was generally smaller than that under the MLM. In particular, one SNP, Marker6619-75, exhibited the minimum *P*-value under both models and could explain the maximum PVE (20.67%) in the GLM and 16.90% of the PVE in the MLM. The marker was also the only association that remained significant after applying the Bonferroni correction (*P* ≤ 1.08E-5). A summary of the favorable SNP alleles is shown in Table 2 and Fig. 2. The phenotypic effects (*a*_i_) of the eleven SNPs that were consistently associated with WT ranged from 0.04 to 0.33, and all the *a*_i_ values reached the *P* < 0.05 or *P* < 0.01 significance level except for Marker6288-117, Marker9771-143, and Marker6288-36. The alleles Marker6619-75 and Marker 5022-204 expressed the greatest *a*_i_ values, and both could increase the WT by 33.0%.

**Table 2:** Summary of common significant SNPs associated with WT detected by GWAS using two models, MLM and GLM

SLAF tag	SNP position	GLM		MLM		Alleles^c^	*a*_i_^d^
		*P* ^a^	*R* ^2b^	*P* ^*a*^	*R* ^2b^		
**Marker6619**	75	3.35E-07*	20.67	1.55E-04	16.90	Y/**C**	0.33**
Marker6288	117	6.32E-05	18.68	1.58E-04	23.60	**A**/G/R	0.18 ns
Marker9771	143	1.11E-05	19.32	2.28E-04	20.76	**A**/C/M	0.04 ns
**Marker18364**	144	3.20E-05	19.83	2.61E-04	21.64	**A**/T/W	0.28*
**Marker12711**	95	2.73E-04	16.33	3.61E-04	20.72	**A**/G/R	0.27*
**Marker3678**	87	2.96E-05	15.14	5.02E-04	14.50	K/**T**	0.29**
Marker6288	36	8.30E-04	14.22	5.66E-04	19.76	**A**/G/R	0.18 ns
**Marker99922**	41	7.08E-04	13.98	6.03E-04	21.85	**G**/A/R	0.26**
**Marker5022**	204	3.58E-05	14.18	8.46E-04	12.85	Y/**C**	0.33**
Marker46623	40	1.01E-03	17.84	9.22E-05	18.36	G/**A**/R	0.21**
Marker5233	18	1.29E-04	12.53	9.21E-04	12.68	R/**G**	0.22**

Letters in bold indicate the significant SNPs used for the pyramiding analysis

^a*^ Markers significantly associated with WT according to the Bonferroni threshold (*P* ≤ 1.08E-05)

^b^*R*^2^ phenotypic variation explanation (PVE)

^c^Minor and major alleles. Favorable alleles are in bold. Degenerate base Y: C/T; R: A/G; M: A/C; W: A/T; K: T/G

^d^*a*_i_ represents phenotypic effects. *P < 0.05 **P < 0.01 ns not significant

### Pyramiding effects of highly favorable SNP alleles

To determine whether the WT-favorable SNP alleles had pyramiding effects, the following six SNP loci exhibiting significant phenotypic effects (*a*_*i*_ values of greater than 0.25), Marker6619-75, Marker18364-144, Marker12711-95, Marker3678-87, Marker99922-41, and Marker5022-204, were selected for further analysis, and the favorable alleles of these loci were C, A, A, T, G, and C, respectively (Table 2). Based on the genotype data, the 88 accessions were grouped into seven classes that contained 0–6 highly favorable alleles, and the mean MFVW values of the different groups were analyzed by ANOVA (Table 3). The results indicated that the more favorable the alleles were, the higher the MFVW values (with more resistance to waterlogging), with the exception being that the MFVW of accessions harboring 5 favorable alleles (0.89) was slightly higher than that with 6 favorable alleles (0.88). This phenomenon may be explained by the lack of a sufficient number of individuals in the panel to appropriately represent these two groups (each contains one entry). Nonetheless, the MFVW of the accessions that held one favorable allele can be significantly increased by 48.89% compared with those that held no favorable alleles (*P* < 0.05). Similarly, the MFVW of the accessions that held two favorable alleles increased by 15.09% compared with those that held only one favorable allele (*P* > 0.05). When the number of favorable alleles reached three, the MFVW of the accessions (0.61) could exceed the mean values of the entire panel (0.58).

**Table 3:** Pyramiding effects of the highly favorable alleles that contribute to WT

No. of favorable alleles	Mean ± *SD*	Frequency/%
0	0.23 ± 0.08 (Dd)	7.95
1	0.45 ± 0.13 (CDc)	4.55
2	0.53 ± 0.18 (BCbc)	9.09
3	0.61 ± 0.13 (BCbc)	68.18
4	0.72 ± 0.17 (ABab)	7.95
5	0.89 ± 0.00 (Aa)	1.14
6	0.88 ± 0.00 (Aa)	1.14

Uppercase letters represent significance at *P* < 0.01, and lowercase letters represent significance at *P* < 0.05

**Fig. 3:**
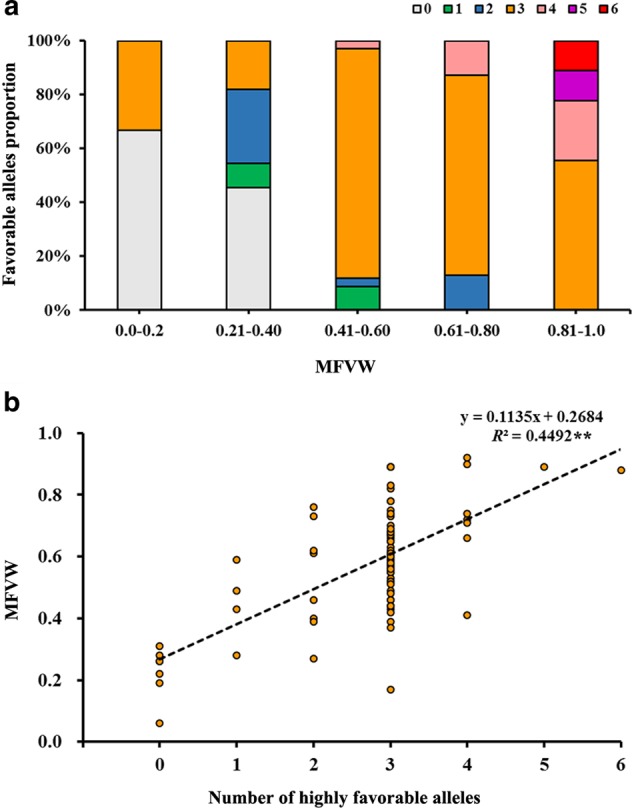
Charts of seven types of genotype accounting for the proportion in five WT grades (**a**) and a linear regression analysis showing the number of favorable alleles and MFVWs (**b**)

Additionally, the accessions that held three favorable alleles accounted for a large proportion of the whole panel; however, the proportion of higher MFVWs was larger than that of lower MFVW values. The accessions holding less than one favorable allele were not found among the chrysanthemum varieties with MFVWs > 0.40, whereas the accessions holding more than three favorable alleles primarily or completely presented MFVWs > 0.81 (Fig. 3a). Furthermore, to assess the pyramiding effects of the highly favorable SNP alleles on the WT, a linear regression analysis was conducted between the number of favorable alleles and the average MFVWs. As a result, a significant positive correlation was observed using the equation *y* = 0.1135 × + 0.2684 (*R*^2^ = 0.45, *P* < 0.01; Fig. 3b), which confirms the existence of pyramiding effects in the favorable alleles. All the above results revealed that the genetic control of WT exhibits a largely additive effect in chrysanthemums.

### Development and verification of the dCAPS marker

**Fig. 4:**
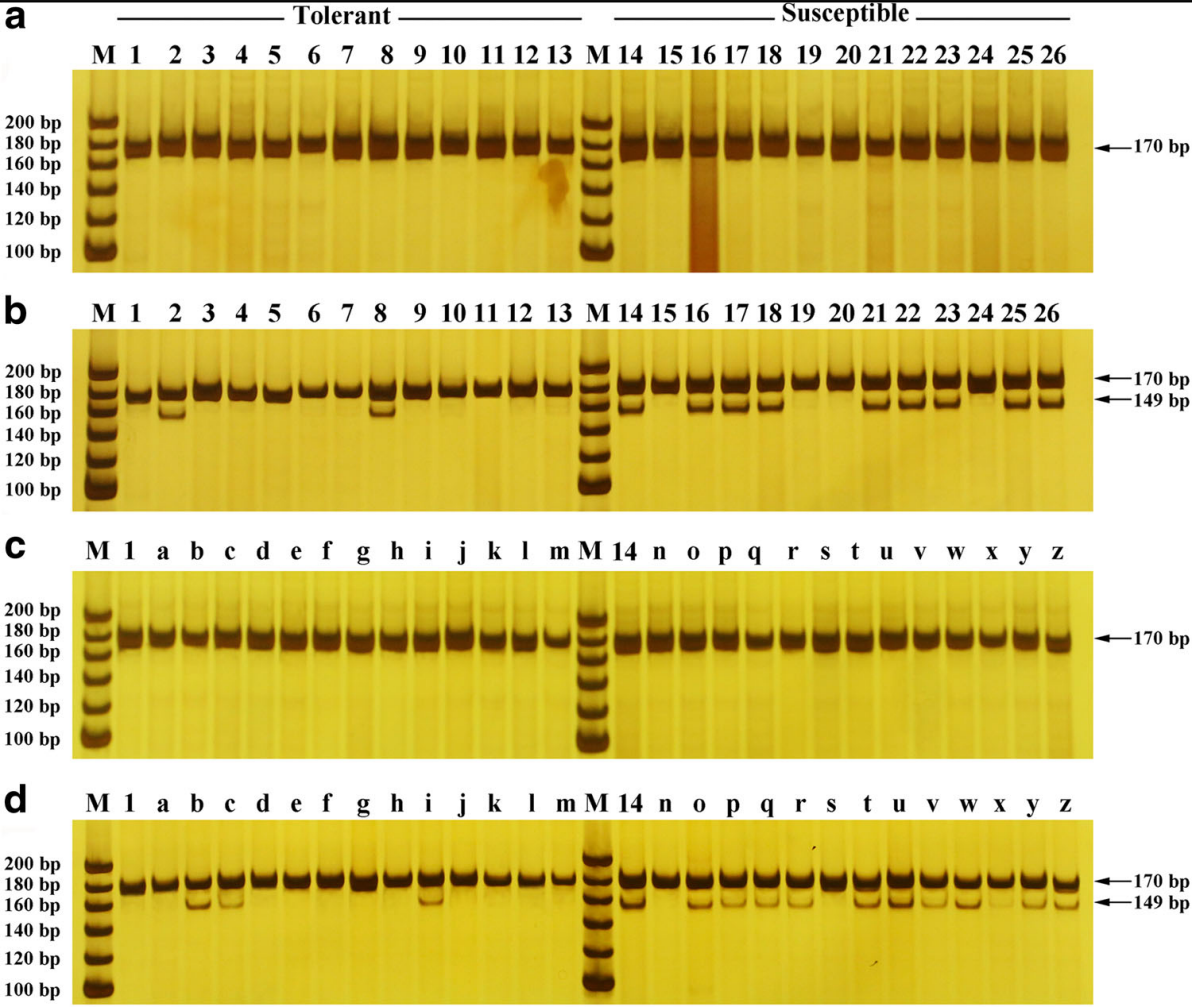
Polyacrylamide gel electrophoresis for undigested (**a**, **c**) and digested (**b**, **d**) WT-dCAPS1 marker products. Lanes 1–13 represent thirteen tolerant accessions, and lanes 14–26 represent thirteen sensitive accessions in a natural population. Lane 1 and lane 14 refer to ‘Nannong Xuefeng’ and ‘Monalisa’, respectively. Lanes a–m represent 13 tolerant F_1_ lines. Lanes n–z represent 13 sensitive F_1_ lines, which were derived from ‘Nannong Xuefeng’ × ‘Monalisa’. M, molecular weight size marker with a 20 bp ladder

Because the SNP locus Marker6619-75 showed a relatively strong association with WT and it had the largest phenotypic effect, it was selected to develop a PCR-based marker for MAS breeding. To develop specific CAPS/dCAPS markers, we introduced a 0–1 mismatch into the forward or reverse primer, and only the commonly used restriction enzymes were considered to design the primers in this study. As a result, a dCAPS marker based on the restriction enzyme *Nhe* I was developed (and termed WT-dCAPS1). The detailed primer designation and sequence information for SLAF tag Marker6619 are shown in Supplementary Table S5 and Supplementary Figure S4. To assess the consistency rate of the WT-dCAPS1, this marker was successively employed in a variety population and an F_1_ population derived from the tolerant cultivar Nannong Xuefeng and the sensitive cultivar Monalisa, with each containing 13 waterlogging-tolerant lines and 13 waterlogging-sensitive lines (Supplementary Table S2). A single target fragment of 170 bp was obtained via polyacrylamide gel electrophoresis from the PCR products of all the materials, which indicated that the designed primer was specific and suitable (Fig. 4a, c). For the digestion products, two different types of banding appeared for all the materials, and they showed clear polymorphisms between the tolerant and susceptible plants. Eleven (84.6%) tolerant lines generated a single undigested 170 bp band, whereas 9 (69.2%) susceptible lines generated two bands of 170 bp and 149 bp and an undetectable 21 bp fragment in the variety population (Fig. 4b). Nannong Xuefeng (Lane 1) and Monalisa (Lane 14) showed different band types as expected, and we also observed a similar result when we verified the WT-dCAPS1 in 26 F_1_ progenies, for 10 (76.9%) tolerant lines that generated a single undigested 170 bp band, whereas 11 (84.6%) susceptible lines showed two bands of 170 bp and 149 bp (Fig. 4d). The result of the restriction endonuclease digestion was in accordance with that anticipated according to the favorable allele and the recognition site. Thus, the estimated consistency rates of the WT-dCAPS1 were 76.9% and 80.8% in the variety and F_1_ populations, respectively, with an average of 78.9%. Moreover, significant correlations at *r* *=* 0.61 and 0.57 in the variety and F_1_ populations, respectively, were detected between the genotype and phenotype for WT-dCAPS1 (*P* < 0.01; Supplementary Table S2), which may be related to the contribution of these inconsistent cases to both the complex background of chrysanthemums and the complex inheritance of the WT trait. Nevertheless, the newly developed dCAPS marker represented a positive attempt to exploit a codominant molecular marker that cosegregated with WT in chrysanthemum.

### Prediction of candidate genes

All SLAF sequences harboring one or more of the 140 SNPs detected under the GLM or MLM models were aligned with the available chrysanthemum transcriptome databases, and only four candidate genes were implicated for their involvement in abiotic stress responses according to the gene annotation (Table 4). The SLAF tag Marker384075 lay within a gene (*CL5639.Contig3_All*) encoding betaine aldehyde dehydrogenase (BADH). Three identified SLAF sequences lay within genes (*Unigene6108_All*, *Unigene33415_All*, and *Unigene21682_All*) encoding three protein kinases, namely APK1B (*Arabidopsis* protein kinase 1), SnRK2.6 (SNF1-related protein kinase 2), and BAM1, respectively. The primer design failed for *Unigene33415_All* as annotated to SnRK2.6 because of the short sequence. The SLAF tag Marker6619 lay within a gene homologous to *Cynara cardunculus* var. *scolymus* (*CL17968.Contig2_All*) that encodes the hypothetical protein Ccrd_010998; however, little is known about its function in plants. Because of the importance of SNP locus Marker6619-75, all four selected candidate genes along with *CL17968.Contig2_All* were preliminarily verified using qRT-PCR, and the relative expression levels are demonstrated in Supplementary Figure S5. The candidate genes showed different degrees of responses to waterlogging stress in the two cultivars with contrasting WT. Notably, the expression of *Unigene6108_All* in the susceptible ‘Monalisa’ cultivar showed a significant increase after exposure to waterlogging stress (70.15%), while no significant increase was found in the tolerant cultivar ‘Nannong Xuefeng’ (14.40%). The expression of *Unigene21682* decreased in the tolerant variety ‘Nannong Xuefeng’ (51.93%), whereas it increased in the susceptible variety ‘Monalisa’ (45.60%). In addition, a similarly decreased expression was found for *CL5639.Contig3_All* and *CL17968.Contig2_All* but did not reach statistical significance.

**Table 4:** List of candidate genes associated with WT

SLAF tag	Position/bp	Candidate gene^a^	Functional annotation
Marker384075	137/179	*CL5639.Contig3_All*	Betaine aldehyde dehydrogenase BADH
Marker28685	14/57	*Unigene6108_All*	Protein kinase APK1B
Marker5356	9/141	*Unigene33415_All*	Serine/threonine-protein kinase SRK2E; Serine/threonine-protein kinase OST1; SNF1-related kinase 2.6 SnRK2.6
Marker4335	53	*Unigene21682_All*	Leucine-rich repeat receptor-like serine/threonine-protein kinase BAM1
Marker6619	75	*CL17968.Contig2_All*	Hypothetical protein Ccrd_010998 [*Cynara cardunculus* var. *scolymus*]

^a^First four candidate genes have been reported to be related to abiotic stress; the function of *CL17968.Contig2_All*, which was also selected to be verified via qRT-PCR, is unknown

## Discussion

The cultivated chrysanthemum is an allohexaploid species with a large and complex genetic background. A lack of genomic information delays the performance of chrysanthemum molecular genetic analyses and breeding, particularly genetic mapping. To date, AFLP, RAPD, ISSR, SRAP, and SSR have been the most commonly used molecular markers in chrysanthemums^20,39,43–45^. However, with the emergence and application of high-throughput NGS technologies, a large number of SNPs have been detected in chrysanthemums and have been made available for further research and breeding^21,46,47^.

GWAS is a useful tool for identifying the genetic loci and candidate genes responsible for the natural variations in a targeted quantitative trait. The power to detect associated loci for a target trait via GWAS typically relies on the marker density, population size, target trait, phenotypic evaluation, and statistical method^48^. Previously, we performed WT association mapping using 707 markers based on SRAP, SCoT, and SSR, and we detected 31 markers related to the WT under the MLM + Q model at a threshold of *P* < 0.01^7^. In the present study, however, a considerable number of SNPs (92,811) were used, and more than 700 loci were identified using the same model and threshold value (data not shown). Long and Langley (1999) showed that increases in the population size have a greater impact on the power of QTL detection than increases in the marker density^49^. Zhu et al.^50^ also indicated that using a larger population would facilitate the exploration of smaller-effect QTLs. Population sizes ranging from 100 to 300 have been frequently documented in GWAS^51–53^; however, this selection also depends on many factors, including the plant species, estimated cost, analysis platform, and objective traits. A large population is not always practicable for GWASs, especially for certain tolerance traits, which often require complicated treatments and the simultaneous measurements of all the lines to obtain accurate phenotypic data. In our study, although an association population with only 88 accessions was not large enough, a moderately large phenotypic variation coefficient (30.74%) was calculated for the MFVW (Supplementary Figure S1), and a low level of relatedness was observed in the population, with kinship values ranging from 0 to 0.447 (with a mean of 0.011) and ~93.94% of the comparisons presenting a coefficient of <0.05 (Supplementary Figure S2d). All the above points supported the success of the association genetic approach used in this study. In addition, several GWAS have been successfully conducted using a small population containing less than 100 entries. For example, Zhang et al. identified 16 SNP loci and eight candidate genes that were significantly associated with drought tolerance by performing a GWAS in a panel of 66 canola accessions^54^. Schulz et al. conducted a GWAS focusing on the anthocyanin and carotenoid contents of petals from 96 diverse rose genotypes^18^.

Repeating experiments during different years or in different places is critical for GWASs, especially for certain complex quantitative traits that are controlled by multiple genes and environmental factors, such as the WT. The stable associations for target traits are more useful in breeding programs with broad adaptability to different environments. Our previous study found that the MFVWs derived from field data were generally lower than those derived from greenhouse-based data, and the two greenhouse experiments exhibited a closer correlation^7^. Hence, another greenhouse pot experiment was repeated in this study. Because the three pot experiments showed strong correlations, with *r* values ranging from 0.61 to 0.74, and no significant environment effect was observed, the average MFVWs of the three trials were used for the GWAS analysis (Supplementary Figure S1). The statistical method is also a determinant that affects the power of the GWAS. The MLM statistical model is more stringent than the GLM model and allows for a large reduction in spurious associations by simultaneously estimating the population structure and the unequal relatedness among individuals. Thus, the MLM model has been popular for use in plant GWASs. In the present study, 137 and 14 SNPs were identified as being significantly associated with WT based on the GLM and MLM models, respectively (*P* ≤ 1E-3). Among these SNPs, 11 were found in both models, which validates the efficiency of GWASs when using an MLM model based on abundant SNP markers compared with gel-based markers. Of particular interest, the SNP locus Marker6619-75 exhibited the minimum *P*-value under both models and presented a comparatively large PVE of 20.67% in the GLM and 16.90% in the MLM. Thus, this locus was selected as an example for developing a PCR marker to assist in breeding.

Associations with higher phenotypic effects for target traits are usually more useful in breeding programs. The favorable alleles and unfavorable alleles are easily determined without considering the heterozygous SNPs in certain species with simple genetic backgrounds^6,55^. Wu et al. set the heterozygous SNP to missing and only used the homozygous SNPs for a GWAS study in *Brassica napus*^56^. The chrysanthemum is a highly heterozygous ornamental species with a complex background, and the heterozygous loci are too common to be neglected. A locus was considered to be in a heterozygous state if the depth of the minor allele was larger than one-third of the total sample depth during SNP calling^21^. The 11 significant SNP loci associated with WT were all heterozygous, and the average MFVW of entries harboring heterozygous alleles was closer to or even lower than the entries harboring unfavorable homozygous alleles for all the associations except for Marker9771-143 (Supplementary Table S7). For example, the average MFVW of accessions with favorable alleles, heterozygous alleles, and unfavorable alleles at Marker99922-41 were 0.81, 0.60, and 0.55, respectively; and the average MFVW of accessions with heterozygous alleles at Marker18364-144 was 0.41, which is significantly lower than that of the accessions with unfavorable homozygous accessions (0.60; *P* < 0.05). As a result, SNP alleles with higher MFVWs that increase the WT were defined as “favorable alleles”, whereas SNP alleles with lower MFVWs, including heterozygous sites, were defined as “unfavorable alleles” in our study. This classification may enlarge the phenotypic effect (*a*_i_) of the favorable alleles for WT.

To date, the effectiveness of marker-based gene pyramiding strategies has been demonstrated in several studies, such as studies on the early maturity and lint percentage traits in upland cotton^55,57^, stigma traits in rice^52^, and clubroot resistance in rapeseed^58^. In our study, six elite alleles that could significantly improve the WT by 25% were mined from the 11 associated loci. Among these alleles, 54.39% were carried by accessions collected from Asia, which presented ~3.1 alleles on average, per entry; 26.36% were carried by accessions from Europe, which presented ~2.1 alleles on average, per entry; and the remaining 20.50% were carried by accessions of unknown provenance. These results suggested that during the chrysanthemum evolution process from Asia to Europe, changes in certain SNP alleles likely resulted in changes in the WT traits. For example, all the unfavorable alleles of Marker6619-75 and Marker 5022-204 were common among the European accessions but were not found in the Asian accessions. Moreover, dosage-pyramiding effects were found in the highly favorable alleles, and the results revealed that the genetic control of WT exhibits a largely additive effect in chrysanthemums (Fig. 3; Table 3), which was also observed in our recent study^39,59^. Finally, nine cross combinations that could potentially pyramid all six selected elite alleles are proposed, including nine waterlogging-tolerant cultivars harboring 3 to 6 favorable alleles (Supplementary Table S6). Interestingly, ‘Qx097’ and ‘Nannong Xuefeng’, which are typical cultivars that possessed 5 and 6 favorable alleles, respectively, were also identified as potential donors for improving the WT in our previous study^7^. These results provide a theoretical basis for proposing a breeding strategy via the polymerization of multiple loci through crossing.

The availability of molecular markers linked specifically to the target trait can facilitate the shortening of the breeding process. Although many molecular marker loci have been identified as being associated with horticultural and tolerances traits in chrysanthemums^7,20,44,60–62^, they cannot be easily applied to directly guide breeding. Zhao et al. transferred a RAPD marker linked to the gene control of the creeping habit of ground-cover chrysanthemums into a superior SCAR (sequence-characterized amplified regions) marker^63^. A simple and low-cost marker is more promising and practical, especially for the selection of early breeding work; therefore, the most prominent SNP locus Marker6619-75 was transformed into a dCAPS marker, which is codominant, locus-specific, and feasible for use in a typical molecular biology laboratory. The WT-dCAPS1 was coassociated with WT at an average efficiency of 78.9%, which is lower than the values reported in other traits and crops^30,64,65^. The inconsistent conditions may be caused by the complexity of the chrysanthemum’s genetic background and WT. Nevertheless, to our knowledge, this study presents the first report of a dCAPS marker linked to WT in plants, and the developed marker validates the method for screening for tolerant germplasm resources and provides a powerful MAS tool. Developing additional dCAPS markers linked to WT and validating them in a larger population are necessary in further studies.

Finally, four candidate genes that encode BADH and the three protein kinases SnRK2, APK1B, and BAM1, as well as one gene with unknown function were predicted via an alignment with the transcriptome library. Plants can activate their signal transduction system when adverse environmental stress occurs. Plant signal transduction can perceive and transduce different stress signals and activate a variety of physiological and biochemical reactions to survive. Plant protein kinases play important roles in signal transduction, growth, development, and gene expression regulation^66^. BADH is one of the two enzymes that catalyze glycine betaine synthesis in chloroplasts from choline, and several studies have reported that *BADH* gene-transgenic plants could enhance tolerance by accumulating glycine betaine, which is an important osmoprotectant that is widely distributed in higher plants and provides protection against various types of abiotic stress by stabilizing the quaternary structure of complex proteins^67^, thereby maintaining the osmotic potential balance with the environment^68^ and stabilizing the oxygen-evolving photosystem II^67^. With respect to WT, Yan et al. observed stimulated *BADH* expression after 1 d of waterlogging stress but the inhibition of the *BADH* expression at 2 d and 4 d in wheat^69^. Similar results were obtained in cotton^70^. APK1 (*Arabidopsis* protein kinase 1) purified from an overproducing *E. coli* strain showed serine/threonine kinase activity and has been shown to be important in mediating stomatal responses to environmental stimuli, including the light intensity, atmospheric carbon dioxide concentration, and the drought hormone abscisic acid^71^. A study showed that the *Arabidopsis APK1b* gene is predominantly expressed in guard cells, and it has an enhanced ability to increase water use efficiency and tolerance to drought conditions^72^. The SnRK2 (SNF1-related protein kinase 2) protein kinase family is unique in plants and consists of 10 members (SnRK2.1 to −2.10) in *Arabidopsis*^73^. Several SnRK2s have been reported to be major kinases in osmotic stress and ABA signaling, which regulates various environmental stresses and developmental and physiological processes. *SRK2E*/*OST1*/*SnRK2.6* encodes an *Arabidopsis* SnRK2 protein kinase and has been found to act as a positive regulator in water stress signaling and ABA-induced stomatal closure^74^. BAM1 is a plastid-targeted *β*-amylase of *Arabidopsis thaliana* that is specifically activated by its reducing conditions, and it plays a role in starch degradation in guard cells to sustain stomatal opening during the day. Several studies in different plant species have indicated that BAM1 regulates a wide variety of developmental^75^ and abiotic stress-related processes, particularly those relating to drought^76^, cold^77^, salt^78^, osmotic^79^, and heat stress^80^. To date, no reports have directly addressed the function of three other candidate genes in waterlogging stress. However, the regulatory networks of various abiotic stresses are fairly complex, and different types of stresses have certain cross interactions. A preliminary qRT-PCR verification experiment was performed in the present study; however, further validation with additional genotypes with contrasting WT traits and additional waterlogging stresses would be required to identify the function of these candidate genes in chrysanthemums.

## Conclusions

A substantial number of SNP markers were used in a GWAS to investigate the genetic control of WT in chrysanthemums. Eleven significant associations and four candidate genes were detected for WT. The dosage-pyramiding effects of the six highly favorable SNP alleles strongly suggests that the WT is largely controlled by quantitative genes with additive effects. Moreover, a major SNP locus (Marker6619-75) was converted into a dCAPS marker, which would be beneficial for future MAS for WT. The results of this study demonstrate that GWASs based on high-density SNP markers represent a powerful approach for dissecting complex WT traits and identifying candidate genes in chrysanthemums.

## Electronic supplementary material

Table S1

Table S2

Table S3

Table S4

Table S5

Table S6

Table S7

Figure S1

Figure S2

Figure S3

Figure S4

Figure S5

## References

[1] Teixeira da Silva JA (2013). Chrysanthemum biotechnology: Quo vadis?. Crit. Rev. Plant Sci..

[2] Yin DM, Chen SM, Chen F, Guan ZY, Fang WM (2009). Morphological and physiological responses of two chrysanthemum cultivars differing in their tolerance to waterlogging. Environ. Exp. Bot..

[3] Zhang X (2013). Identification of major QTL for waterlogging tolerance using genome-wide association and linkage mapping of maize seedlings. Plant. Mol. Biol. Rep..

[4] Valliyodan B (2017). Genetic diversity and genomic strategies for improving drought and waterlogging tolerance in soybeans. J. Exp. Bot..

[5] Zhang X (2017). A new major-effect QTL for waterlogging tolerance in wild barley (*H*. *spontaneum*). Theor. Appl. Genet..

[6] Soltani A (2017). Genetic architecture of flooding tolerance in the dry bean middle-american diversity panel. Front. Plant Sci..

[7] Su J (2016). Genetic variation and association mapping of waterlogging tolerance in chrysanthemum. Planta.

[8] Mccouch SR (2010). Development of genome-wide SNP assays for rice. Breed. Sci..

[9] Baird NA (2008). Rapid SNP discovery and genetic mapping using sequenced RAD markers. PLoS ONE.

[10] Elshire RJ (2011). A robust, simple genotyping-by-sequencing (GBS) approach for high diversity species. PLoS ONE.

[11] Sun X (2013). SLAF-seq: an efficient method of large-scale de novo SNP discovery and genotyping using high-throughput sequencing. PLoS ONE.

[12] Dacosta JM, Sorenson MD (2014). Amplification biases and consistent recovery of loci in a double-digest RAD-seq protocol. PLoS ONE.

[13] Agarwal M, Shrivastava N, Padh H (2008). Advances in molecular marker techniques and their applications in plant sciences. Plant Cell Rep..

[14] Huang X (2010). Genome-wide association studies of 14 agronomic traits in rice landraces. Nat. Genet..

[15] Horton MW (2014). Genome-wide association study of *Arabidopsis thaliana* leaf microbial community. Nat. Commun..

[16] Revilla P (2016). Association mapping for cold tolerance in two large maize inbred panels. BMC Plant Biol..

[17] Zanke CD (2015). Analysis of main effect QTL for thousand grain weight in European winter wheat (*Triticum aestivum* L.) by genome-wide association mapping. Front. Plant Sci..

[18] Schulz DF (2016). Genome-wide association analysis of the anthocyanin and carotenoid contents of rose petals. Front. Plant Sci..

[19] Klie M, Menz I, Linde M, Debener T (2016). Strigolactone pathway genes and plant architecture: association analysis and QTL detection for horticultural traits in chrysanthemum. Mol. Genet. Genom..

[20] Li P (2016). Genetic diversity, population structure and association analysis in cut chrysanthemum (*Chrysanthemum morifolium* Ramat.). Mol. Genet. Genom..

[21] Chong X (2016). A SNP-enabled assessment of genetic diversity, evolutionary relationships and the identification of candidate genes in chrysanthemum. Genome Biol. Evol..

[22] Bertin I, Zhu JH, Gale MD (2005). SSCP-SNP in pearl millet—a new marker system for comparative genetics. Theor. Appl. Genet..

[23] Lehmensiek A, Sutherland MW, Mcnamara RB (2008). The use of high resolution melting (HRM) to map single nucleotide polymorphism markers linked to a covered smut resistance gene in barley. Theor. Appl. Genet..

[24] Lei TG (2012). Development of CAPS markers and allele-specific PCR primers in citrus. Acta Hortic. Sin..

[25] Lestari P, Koh HJ (2013). Development of new CAPS/dCAPS and SNAP markers for rice eating quality. Hayati J. Biosci..

[26] Semagn K, Babu R, Hearne S, Olsen M (2014). Single nucleotide polymorphism genotyping using kompetitive allele specific PCR (KASP): overview of the technology and its application in crop improvement. Mol. Breed..

[27] Neff MM, Turk E, Kalishman M (2002). Web-based primer design for single nucleotide polymorphism analysis. Trends Genet..

[28] Yanagisawa T, Kiribuchi-Otobe C, Hirano H, Suzuki Y, Fujita M (2003). Detection of single nucleotide polymorphism (SNP) controlling the waxy character in wheat by using a derived cleaved amplified polymorphic sequence (dCAPS) marker. Theor. Appl. Genet..

[29] De Castro AP, Blanca JM, Díez MJ, Vinals FN (2007). Identification of a CAPS marker tightly linked to the Tomato yellow leaf curl disease resistance gene *Ty-1* in tomato. Eur. J. Plant Pathol..

[30] Di H (2015). Development of SNP-based dCAPS markers linked to major head smut resistance quantitative trait locus qHS2.09 in maize. Euphytica.

[31] Kushanov FN (2016). Development, genetic mapping and QTL association of cotton *PHYA*, *PHYB*, and *HY5*-specific CAPS and dCAPS markers. BMC Genet..

[32] Saitou N, Nei M (1987). The neighbor-joining method: a new method for reconstructing phylogenetic trees. Mol. Biol. Evol..

[33] Tamura K (2011). MEGA5: molecular evolutionary genetics analysis using maximum likelihood, evolutionary distance, and maximum parsimony methods. Mol. Biol. Evol..

[34] Alexander DH, Novembre J, Lange K (2009). Fast model-based estimation of ancestry in unrelategd individuals. Genome Res..

[35] Hardy OJ, Vekemans X (2002). SPAGeDi: a versatile computer program to analyse spatial genetic structure at the individual or population levels. Mol. Ecol. Resour..

[36] de Hoon MJ, Imoto S, Nolan J, Miyano S (2004). Open source clustering software. Bioinformatics.

[37] Bradbury PJ (2007). TASSEL: software for association mapping of complex traits in diverse samples. Bioinformatics.

[38] Kan G (2015). Association mapping of soybean seed germination under salt stress. Mol. Genet. Genom..

[39] Su J (2018). Dynamic and epistatic QTL mapping reveals the complex genetic architecture of waterlogging tolerance in chrysanthemum. Planta.

[40] Murray MG, Thompson WF (1980). Rapid isolation of high molecular weight plant DNA. Nucleic Acids Res..

[41] Cheng P (2018). A transcriptomic analysis targeting genes involved in the floral transition of winter-flowering chrysanthemum. J. Plant. Growth Regul..

[42] Livak KJ, Schmittgen TD (2012). Analysis of relative gene expression data using real-time quantitative PCR and the 2^−ΔΔCT^ method. Methods.

[43] Zhang F, Chen S, Chen F, Fang W, Li F (2010). A preliminary genetic linkage map of chrysanthemum (*Chrysanthemum morifolium*) cultivars using RAPD, ISSR and AFLP markers. Sci. Hortic..

[44] Zhang F (2011). SRAP-based mapping and QTL detection for inflorescence-related traits in chrysanthemum *(Dendranthema morifolium*). Mol. Breed..

[45] Wang C (2014). Inheritance and molecular markers for aphid (*Macrosiphoniella sanbourni*) resistance in chrysanthemum (*Chrysanthemum morifolium* Ramat.). Sci. Hortic..

[46] van Geest G (2017). An ultra-dense integrated linkage map for hexaploid chrysanthemum enables multi-allelic QTL analysis. Theor. Appl. Genet..

[47] van Geest G (2017). Conclusive evidence for hexasomic inheritance in chrysanthemum based on analysis of a 183 k SNP array. BMC Genom..

[48] Zhang J (2015). Identification of putative candidate genes for water stress tolerance in canola (*Brassica napus*). Front. Plant Sci..

[49] Long AD, Langley CH (1999). The power of association studies to detect the contribution of candidate genetic loci to variation in complex traits. Genome Res..

[50] Zhu C, Gore M, Buckler ES, Yu J (2008). Status and prospects of association mapping in plants. Plant Genome.

[51] Upadhyaya HD, Wang YH, Gowda CLL, Sharma S (2013). Association mapping of maturity and plant height using SNP markers with the sorghum mini core collection. Theor. Appl. Genet..

[52] Dang X (2016). QTL detection and elite alleles mining for stigma traits in *Oryza sativa* by association mapping. Front. Plant Sci..

[53] Zhou Q (2017). Genome-wide SNP markers based on SLAF-Seq uncover breeding traces in rapeseed (*Brassica napus* L.). Front. Plant Sci..

[54] Zhang J (2015). Genome-wide association mapping for tomato volatiles positively contributing to tomato flavor. Front. Plant Sci..

[55] Su J (2016). Detection of favorable QTL alleles and candidate genes for lint percentage by GWAS in Chinese upland cotton. Front. Plant Sci..

[56] Wu J (2016). Genome-wide association study identifies new loci for resistance to sclerotinia stem rot in *Brassica napus*. Front. Plant Sci..

[57] Su J (2016). Identification of favorable SNP alleles and candidate genes for traits related to early maturity via GWAS in upland cotton. BMC Genom..

[58] Li L (2016). A genome-wide association study reveals new loci for resistance to clubroot disease in *Brassica napus*. Front. Plant Sci..

[59] Su J (2017). Combining ability, heterosis, genetic distance and their intercorrelations for waterlogging tolerance traits in chrysanthemum. Euphytica.

[60] Zhang F, Jiang J, Chen S, Chen F, Fang W (2012). Detection of quantitative trait loci for leaf traits in chrysanthemum. J. Hortic. Sci. Biotech..

[61] Zhang F, Jiang J, Chen S, Chen F, Fang W (2012). Mapping single-locus and epistatic quantitative trait loci for plant architectural traits in chrysanthemum. Mol. Breed..

[62] Fu X (2018). Genetic variation and association mapping of aphid (*Macrosiphoniella sanbourni*) resistance in chrysanthemum (*Chrysanthemum morifolium* Ramat.). Euphytica.

[63] Zhao J, Chen S, Chen F (2009). Conversion of RAPD marker linked to creep plant type in ground-cover chrysanthemum to SCAR marker. Sci. Silvae Sin..

[64] Shi XH (2015). Development and utilization of CAPS/dCAPS markers based on the SNPs lying in soybean cyst nematode resistant genes Rhg4. Acta Agron. Sin..

[65] Zhu WW (2015). Development and verification of a CAPS marker linked to tuber shape gene in potato. Acta Agron. Sin..

[66] Stone JM, Walker JC (1995). Plant protein kinase families and signal transduction. Plant Physiol..

[67] Murata N, Mohanty PS, Hayashi H, Papageorgiou GC (1992). Glycinebetaine stabilizes the association of extrinsic proteins with the photosynthetic oxygen‐evolving complex. FEBS Lett..

[68] Robinson SP, Jones GP (1986). Accumulation of glycinebetaine in chloroplasts provides osmotic adjustment during salt stress. Funct. Plant Biol..

[69] Yan JP, Liang Y, Tan XL (2011). Expression of *BADH* in young root of wheat (*Triticum aestivum*) under waterlog and low temperature stress. Hubei Agr. Sci..

[70] Yan JP, Liang Y, Tan XL (2011). Expression of ADHa and BADH in young root of cotton (*Gossypium hirsutum*) under waterlogged stress. China Cotton.

[71] Hirayama T, Oka A (1992). Novel protein kinase of *Arabidopsis thaliana* (APK_1_) that phosphorylates tyrosine, serine and threonine. Plant Mol. Biol..

[72] Elhaddad NS, Hunt L, Sloan J, Gray JE (2014). Light-induced stomatal opening is affected by the guard cell protein kinase APK1b. PLoS ONE.

[73] Hrabak EM (2003). The *Arabidopsis* CDPK-SnRK superfamily of protein kinases. Plant Physiol..

[74] Yoshida R (2006). The regulatory domain of SRK2E/OST1/SnRK2.6 interacts with ABI1 and integrates abscisic acid (ABA) and osmotic stress signals controlling stomatal closure in Arabidopsis. J. Biol. Chem..

[75] Hord CL, Chen C, Deyoung BJ, Clark SE, Ma H (2006). The BAM_1_/BAM_2_ receptor-like kinases are important regulators of *Arabidopsis* early anther development. Plant Cell.

[76] Zanella M (2016). *β*-amylase 1 (BAM1) degrades transitory starch to sustain proline biosynthesis during drought stress. J. Exp. Bot..

[77] Maruyama K (2009). Metabolic pathways involved in cold acclimation identified by integrated analysis of metabolites and transcripts regulated by DREB1A and DREB2A. Plant Physiol..

[78] Jha A, Saxena J, Sharma V (2013). Investigation on phosphate solubilization potential of agricultural soil bacteria as affected by different phosphorus sources, temperature, salt, and pH. Commun. Soil Sci. Plan..

[79] Valerio C (2011). Thioredoxin-regulated *β*-amylase (BAM1) triggers diurnal starch degradation in guard cells, and in mesophyll cells under osmotic stress. J. Exp. Bot..

[80] Monroe JD (2014). *β*-Amylase1 and *β*-Amylase3 are plastidic starch hydrolases in Arabidopsis that seem to be adapted for different thermal, pH, and stress conditions. Plant Physiol..

