# Supplementary material for: Genome-wide association study identifies favorable SNP alleles and candidate genes for waterlogging tolerance in chrysanthemums
Source: Hortic Res. 2019 Feb 1;6:21. doi: 10.1038/s41438-018-0101-7 (PMC6355785; doi:10.1038/s41438-018-0101-7)
Supplement: Supplementary file 1 — Table S1 [file 41438_2018_101_MOESM1_ESM.docx]

**Table S1** Eighty-eight entries composing the GWAS germplasm panel along with their origin, MFVW and WT grades in the three pot experiments

| Code | Accessions | Type | Origin | MFVW ^a^ | | | | Grade ^b^ |
| --- | --- | --- | --- | --- | --- | --- | --- | --- |
|  |  |  |  | EXP.1 | EXP.2 | EXP.3 | mean |  |
| fx | Jinba | Disbud | Japan | 0.50 | 0.72 | 0.72 | 0.65 | Ⅱ |
| ej | Jingyun | Disbud | Japan | 0.89 | 0.91 | 0.65 | 0.82 | Ⅰ |
| hn | Youxiang | Disbud | Japan | 0.60 | 0.75 | 0.71 | 0.69 | Ⅱ |
| fm | Qd005 | Disbud | Japan | 0.48 | 0.55 | 0.57 | 0.53 | Ⅲ |
| fq | Jinghai | Disbud | Japan | 0.55 | 0.55 | 0.47 | 0.52 | Ⅲ |
| cx | Autumn Yellow | Disbud | Japan | 0.70 | 0.56 | 0.45 | 0.57 | Ⅲ |
| cn | Qd011 | Disbud | China | 0.59 | 0.71 | 0.89 | 0.73 | Ⅱ |
| az | The Wood | Disbud | Japan | 0.41 | 0.71 | 0.55 | 0.56 | Ⅲ |
| ee | Shunfa | Disbud | China | 0.66 | 0.89 | 0.74 | 0.76 | Ⅱ |
| ao | Qd021 | Disbud | unknown | 0.49 | 0.70 | 0.71 | 0.63 | Ⅱ |
| av | Guangju | Disbud | China | 0.69 | 0.67 | 0.41 | 0.59 | Ⅲ |
| ga | Jiuyuehuang | Disbud | China | 0.65 | 0.77 | 0.63 | 0.68 | Ⅱ |
| dq | Datouhuang | Disbud | China | 0.41 | 0.39 | 0.38 | 0.39 | Ⅳ |
| ar | Xiwang Zhiguang | Disbud | China | 1.00 | 0.94 | 0.72 | 0.89 | Ⅰ |
| hb | Classic | Disbud | Japan | 0.58 | 0.65 | 0.68 | 0.64 | Ⅱ |
| ew | Jinjingjing | Disbud | Japan | 0.96 | 0.89 | 0.63 | 0.83 | Ⅰ |
| en | Cuixin | Disbud | unknown | 0.86 | 0.73 | 0.56 | 0.72 | Ⅱ |
| fn | Charm lane | Disbud | Japan | 0.76 | 0.55 | 0.73 | 0.68 | Ⅱ |
| ac | Lasting Violet | Disbud | Japan | 0.54 | 0.35 | 0.87 | 0.59 | Ⅲ |
| dg | Xiufang Yuan | Disbud | Japan | 0.83 | 0.81 | 0.68 | 0.77 | Ⅱ |
| bn | Monthly Yellow | Disbud | Japan | 0.61 | 0.70 | 0.57 | 0.63 | Ⅱ |
| he | Anna Green | Disbud | Europe | 0.39 | 0.57 | 0.53 | 0.50 | Ⅲ |
| du | Huangqieju | Disbud | China | 0.43 | 0.51 | 0.47 | 0.47 | Ⅲ |
| bf | Golden | Disbud | Japan | 0.60 | 0.73 | 0.63 | 0.65 | Ⅱ |
| cf | Qx001 | Spray | Europe | 0.65 | 0.69 | 0.66 | 0.67 | Ⅱ |
| bh | Grand White | Spray | Europe | 0.26 | 0.57 | 0.37 | 0.40 | Ⅳ |
| hd | Grand Rose. | Spray | Europe | 0.32 | 0.29 | 0.27 | 0.29 | Ⅳ |
| bx | Qx008 | Spray | unknown | 0.73 | 0.50 | 0.45 | 0.56 | Ⅲ |
| hj | Dazzler | Spray | Europe | 0.68 | 0.79 | 0.52 | 0.66 | Ⅱ |
| hg | Qx013 | Spray | unknown | 0.30 | 0.40 | 0.60 | 0.43 | Ⅲ |
| ap | Reddy | Spray | Europe | 0.65 | 0.57 | 0.52 | 0.58 | Ⅲ |
| bj | Xiaoli | Spray | Japan | 0.85 | 0.81 | 1.00 | 0.89 | Ⅰ |
| dh | Huoyan | Spray | Japan | 0.89 | 0.93 | 0.84 | 0.89 | Ⅰ |
| fb | Statesman | Spray | Europe | 0.59 | 0.40 | 0.70 | 0.56 | Ⅲ |
| ge | Feeling White | Spray | Europe | 0.57 | 0.58 | 0.66 | 0.60 | Ⅲ |
| bd | Froggy | Spray | Europe | 0.38 | 0.20 | 0.24 | 0.27 | Ⅳ |
| bm | Cayman | Spray | Europe | 0.26 | 0.50 | 0.53 | 0.43 | Ⅲ |
| am | Qx042 | Spray | unknown | 0.53 | 0.76 | 0.71 | 0.67 | Ⅱ |
| gj | Puma white | Spray | Europe | 0.24 | 0.28 | 0.11 | 0.21 | Ⅳ |
| fg | Qx044 | Spray | unknown | 0.66 | 0.68 | 0.47 | 0.60 | Ⅲ |
| ae | Mundial Improved. | Spray | Europe | 0.38 | 0.25 | 0.20 | 0.28 | Ⅳ |
| db | Finch. | Spray | Europe | 0.86 | 0.55 | 0.62 | 0.68 | Ⅱ |
| dr | Puma Sunny | Spray | Europe | 0.08 | 0.07 | 0.12 | 0.09 | Ⅴ |
| fc | Sunny | Spray | unknown | 0.43 | 0.32 | 0.53 | 0.43 | Ⅲ |
| an | Pinwheel | Spray | Japan | 0.78 | 0.63 | 0.55 | 0.65 | Ⅱ |
| fl | Albert Heijn | Spray | Europe | 0.66 | 0.73 | 0.75 | 0.71 | Ⅱ |
| cz | Daymark Cream | Spray | South Korea | 0.73 | 0.60 | 0.42 | 0.58 | Ⅲ |
| ek | Isis | Spray | South Korea | 0.59 | 0.24 | 0.45 | 0.43 | Ⅲ |
| hc | Qx062 | Spray | Europe | 0.53 | 0.72 | 0.45 | 0.57 | Ⅲ |
| hm | Vyking dark | Spray | Europe | 0.57 | 0.47 | 0.33 | 0.46 | Ⅲ |
| fv | Qx073 | Spray | Japan | 0.36 | 0.45 | 0.35 | 0.39 | Ⅳ |
| aj | Qx074 | Spray | Japan | 0.86 | 0.67 | 0.62 | 0.72 | Ⅱ |
| bu | Winter White | Spray | Japan | 1.00 | 0.88 | 0.78 | 0.89 | Ⅰ |
| gi | Qx083 | Spray | Japan | 0.56 | 0.67 | 0.56 | 0.60 | Ⅲ |
| eb | Qx085 | Spray | Japan | 0.69 | 0.68 | 0.50 | 0.62 | Ⅱ |
| ax | Summer pink | Spray | Japan | 0.32 | 0.23 | 0.00 | 0.18 | Ⅴ |
| eh | Qx091 | Spray | unknown | 0.66 | 0.68 | 0.54 | 0.63 | Ⅱ |
| hr | Qx093 | Spray | unknown | 0.67 | 0.90 | 0.67 | 0.75 | Ⅱ |
| gv | Qx095 | Spray | unknown | 0.77 | 0.71 | 0.54 | 0.67 | Ⅱ |
| go | Qx096 | Spray | unknown | 0.36 | 0.56 | 0.42 | 0.45 | Ⅲ |
| dm | Qx097 | Spray | unknown | 0.84 | 0.88 | 0.94 | 0.89 | Ⅰ |
| eq | Qx098 | Spray | unknown | 0.22 | 0.56 | 0.62 | 0.47 | Ⅲ |
| et | Qx099 | Spray | unknown | 0.70 | 0.81 | 0.75 | 0.75 | Ⅱ |
| cp | Qx106 | Spray | Europe | 0.36 | 0.38 | 0.43 | 0.39 | Ⅳ |
| dn | Winmbledon | Spray | Europe | 0.49 | 0.43 | 0.49 | 0.47 | Ⅲ |
| dy | Monalisa | Spray | Europe | 0.21 | 0.21 | 0.16 | 0.19 | Ⅴ |
| co | Qx114 | Spray | unknown | 0.42 | 0.68 | 0.71 | 0.60 | Ⅲ |
| gk | Qx116 | Spray | unknown | 0.19 | 0.15 | 0.47 | 0.27 | Ⅳ |
| dk | Zembla Sunny | Spray | Europe | 0.62 | 0.63 | 0.60 | 0.62 | Ⅱ |
| cq | Delilah | Spray | Europe | 0.78 | 0.86 | 0.92 | 0.85 | Ⅰ |
| bg | Marimo | Spray | Europe | 0.48 | 0.43 | 0.60 | 0.50 | Ⅲ |
| au | Samos Dark | Spray | Europe | 0.55 | 0.51 | 0.38 | 0.48 | Ⅲ |
| fr | Noa | Spray | Europe | 0.55 | 0.64 | 0.49 | 0.56 | Ⅲ |
| cg | Wimbledon | Spray | Europe | 0.46 | 0.47 | 0.55 | 0.49 | Ⅲ |
| cy | King Fisher | Spray | Europe | 0.53 | 0.70 | 0.61 | 0.61 | Ⅱ |
| ey | Qx147 | Spray | Europe | 0.53 | 0.59 | 0.73 | 0.62 | Ⅱ |
| dd | Qx148 | Spray | China | 0.58 | 0.52 | 0.57 | 0.56 | Ⅲ |
| fz | Red Ping-pong | Spray | China | 0.72 | 0.69 | 0.68 | 0.70 | Ⅱ |
| fp | Qx151 | Spray | China | 0.49 | 0.41 | 0.45 | 0.45 | Ⅲ |
| cc | Monalis Cream | Spray | Europe | 0.38 | 0.16 | 0.41 | 0.32 | Ⅳ |
| ev | Monalisa Currant | Spray | Europe | 0.41 | 0.06 | 0.29 | 0.25 | Ⅳ |
| dv | Nannong Feizi | Spray | China | 0.77 | 0.78 | 0.80 | 0.78 | Ⅱ |
| fh | Nannong Gongxun | Spray | China | 0.73 | 0.63 | 0.37 | 0.58 | Ⅲ |
| de | Nannong Hongfeng | Spray | China | 0.63 | 0.62 | 0.69 | 0.65 | Ⅱ |
| gx | Nannong Hongxia | Spray | China | 0.53 | 0.85 | 0.36 | 0.58 | Ⅲ |
| dc | Nannong Jinguan | Spray | China | 0.64 | 0.57 | 0.59 | 0.60 | Ⅲ |
| gg | Nannong Xuefeng | Spray | China | 0.95 | 0.87 | 0.83 | 0.88 | Ⅰ |
| cs | Nannong Zichun | Spray | China | 0.77 | 0.74 | 0.81 | 0.77 | Ⅱ |

^a^ Data for EXP.1 and EXP.2 were reported in our previous study (Su et al., 2016a);

^b^ I highly tolerant, MFVW > 0.80; II tolerant, 0.60 < MFVW ≤ 0.8; III moderately tolerant, 0.40 < MFVW ≤ 0.60; IV susceptible, 0.20 < MFVW ≤ 0.4; and V highly susceptible, MFVW ≤ 0.2.

|  |
| --- |
|  |
