# Supplementary material for: Genome-wide association study identifies favorable SNP alleles and candidate genes for waterlogging tolerance in chrysanthemums
Source: Hortic Res. 2019 Feb 1;6:21. doi: 10.1038/s41438-018-0101-7 (PMC6355785; doi:10.1038/s41438-018-0101-7)
Supplement: Supplementary file 2 — Table S2 [file 41438_2018_101_MOESM2_ESM.docx]

**Table S2** List of the accessions used for verifying dCAPS marker in this study

| Code | Accession | MFVW ^a^ | Allele ^b^ | Code | Accession | MFVW ^a^ | Allele ^b^ |
| --- | --- | --- | --- | --- | --- | --- | --- |
| *Variety population* | | | | | |  |  |
| 1 | Nannong Xuefeng | 0.88 | F | 14 | Monalisa | 0.19 | U |
| 2 | Nannong Feizi | 0.78 | U | 15 | Nannong Jinningmeng | 0.17 | F |
| 3 | Nannong Zichun | 0.78 | F | 16 | Grand Rose. | 0.29 | U |
| 4 | Xiaoli | 0.89 | F | 17 | Puma white | 0.21 | U |
| 5 | Winter White | 0.89 | F | 18 | Puma Sunny | 0.09 | U |
| 6 | Qx097 | 0.89 | F | 19 | Qx096 | 0.45 | F |
| 7 | Dubin | 0.72 | F | 20 | Qx098 | 0.47 | F |
| 8 | Grand Orange.deep | 0.79 | U | 21 | Euro Sunny | 0.24 | U |
| 9 | Pinwheel | 0.65 | F | 22 | Monalisa Rosy | 0.33 | U |
| 10 | Qx093 | 0.75 | F | 23 | Qx116 | 0.27 | U |
| 11 | Nannong Xunzhang | 0.86 | F | 24 | Angelina | 0.34 | F |
| 12 | Qd008 | 0.91 | F | 25 | Monalis Cream | 0.32 | U |
| 13 | Monthly Yellow | 0.63 | F | 26 | Lvyun | 0.17 | U |
| *F_1_ population* | | | | | |  |  |
| a | 82 | 0.92 | F | n | 155 | 0.12 | F |
| b | 180 | 0.92 | U | o | 134 | 0.14 | U |
| c | 78 | 0.92 | U | p | 16 | 0.14 | U |
| d | 81 | 0.91 | F | q | 79 | 0.25 | U |
| e | 165 | 0.88 | F | r | 70 | 0.18 | U |
| f | 95 | 0.88 | F | s | 205 | 0.25 | F |
| g | 94 | 0.87 | F | t | 41 | 0.2 | U |
| h | 160 | 0.83 | F | u | 11 | 0.26 | U |
| i | 168 | 0.82 | U | v | 200 | 0.23 | U |
| j | 174 | 0.82 | F | w | 24 | 0.17 | U |
| k | 189 | 0.81 | F | x | 61 | 0.27 | U |
| l | 117 | 0.76 | F | y | 111 | 0.27 | U |
| m | 25 | 0.78 | F | z | 39 | 0.34 | U |
| *correlation coefficients* ^c^ | | *r* = 0.61** (variety population) | | | | | |
|  |  | *r* = 0.57** (F_1_ population) | | | | | |

^a^ MFVW (membership function value of waterlogging) is a comprehensive index of WT ranging from 0 to 1, and a higher MFVW is indicative of an enhanced WT (Su et al., 2016a);

^b^ F, favorable allele, U, unfavorable allele;

^c^ The correlation coefficient between the genotype and phenotype for WT-dCAPS1, ** indicates *P* < 0.01.
