# Supplementary material for: Genome-wide association study identifies favorable SNP alleles and candidate genes for waterlogging tolerance in chrysanthemums
Source: Hortic Res. 2019 Feb 1;6:21. doi: 10.1038/s41438-018-0101-7 (PMC6355785; doi:10.1038/s41438-018-0101-7)
Supplement: Supplementary file 3 — Table S3 [file 41438_2018_101_MOESM3_ESM.docx]

**Table S3** Primer sequences for qRT-PCR

| Unigenes | Forward primer (5’- 3’) | Reverse primer (5’- 3’) |
| --- | --- | --- |
| *CL5639.Contig3_All* | AAAGCGTAGTGGGTTTGGTC | ACACTGGACTTCTGCCCTCT |
| *Unigene6108_All* | GGAACCAAAGATGCGACC | ACGGGAACAAGACTGGAAAC |
| *Unigene21682_All* | GGGTTTGGGTAAATGTTCG | CGGTTTTCAGACAGATTCCC |
| *CL17968.Contig2_All* | GTCAAAACTGGTCCTGTCCC | GCCCAAACAAGAACCAGTC |
| *EF1α* | TTTTGGTATCTGGTCCTGGAG | CCATTCAAGCGACAGACTCA |
