# Supplementary material for: Genome-wide association study identifies favorable SNP alleles and candidate genes for waterlogging tolerance in chrysanthemums
Source: Hortic Res. 2019 Feb 1;6:21. doi: 10.1038/s41438-018-0101-7 (PMC6355785; doi:10.1038/s41438-018-0101-7)
Supplement: Supplementary file 5 — Table S5 [file 41438_2018_101_MOESM5_ESM.docx]

**Table S5** Information on WT-dCAPS1 marker developed from Marker6619-75

| Marker | SLAF tag | SNP position | | Primer (5'-3')^a^ | Type ^b^ | Enzyme | Recognition  site | Expected  products/bp ^c^ |
| --- | --- | --- | --- | --- | --- | --- | --- | --- |
| WT_dCAPS1 | Marker6619 | 75 | | F:ATGCACAAGGCATTGGTCTTG*c* | PS | *Nhe* I | G^CTAGC | 170/21+149 |
|  |  |  |  | R:CCTCAAAGTTTTGGCTATTCTCC |  |  |  |  |

^a^ Italicized lowercase letter is a mismatched base;

^b^ PS, the PCR product from WT susceptible accessions could be partially digested;

^c^ Expected product size of the PCR product and after digestion.
