# Supplementary material for: Genome-wide association study identifies favorable SNP alleles and candidate genes for waterlogging tolerance in chrysanthemums
Source: Hortic Res. 2019 Feb 1;6:21. doi: 10.1038/s41438-018-0101-7 (PMC6355785; doi:10.1038/s41438-018-0101-7)
Supplement: Supplementary file 6 — Table S6 [file 41438_2018_101_MOESM6_ESM.docx]

**Table S6** Favorable alleles carried by the superior parents and optimal cross-combination predictions for improving the WT of chrysanthemum

|  | Favorable alleles ^a^ | | | | | |
| --- | --- | --- | --- | --- | --- | --- |
|  | Marker6619-75 | Marker18364-144 | Marker12711-95 | Marker3678-87 | Marker99922-41 | Marker5022-204 |
| *Accessions* |  |  |  |  |  |  |
| Xiaoli | + |  |  | + | + | + |
| Delilah | + | + |  | + |  | + |
| Xiwang Zhiguang | + |  |  | + |  | + |
| Winter White | + |  |  | + |  | + |
| Huoyan | + |  |  | + |  | + |
| Qx097 | + |  | + | + | + | + |
| Nannong Xuefeng | + | + | + | + | + | + |
| Jinjingjing | + |  |  | + |  | + |
| Jingyun | + |  |  | + |  | + |
| *Predicted combinations* | Delilah × Qx097, Nannong Xuefeng × (Xiaoli, Delilah, Xiwang Zhiguang, Winter White, Huoyan, Qx097, Jinjingjing, Jingyun) | | | | | |

^a^ + indicates the favorable alleles
