# Supplementary material for: Genome-wide association study identifies favorable SNP alleles and candidate genes for waterlogging tolerance in chrysanthemums
Source: Hortic Res. 2019 Feb 1;6:21. doi: 10.1038/s41438-018-0101-7 (PMC6355785; doi:10.1038/s41438-018-0101-7)
Supplement: Supplementary file 7 — Table S7 [file 41438_2018_101_MOESM7_ESM.docx]

**Table S7** WT performance of the heterozygous loci at the 11 common significant SNPs

| No. | SLAF tag | SNP position | Allele (MFVW) ^a^ | | |
| --- | --- | --- | --- | --- | --- |
| 1 | Marker6619 | 75 | **C** (0.62Aa) | Y (0.29Bb) |  |
| 2 | Marker6288 | 117 | **A** (0.75Aa) | R (0.37Bb) | G (0.63Aa) |
| 3 | Marker9771 | 143 | **A** (0.61Aa) | M (0.65Aa) | C (0.45Bb) |
| 4 | Marker18364 | 144 | **A** (0.92Aa) | W (0.41Bc) | T (0.60ABb) |
| 5 | Marker12711 | 95 | **A** (0.83Aa) | R (0.66Aab) | G (0.55Ab) |
| 6 | Marker3678 | 87 | **T** (0.61Aa) | K (0.32Bb) |  |
| 7 | Marker6288 | 36 | **A** (0.75Aa) | R (0.40Bb) | G (0.62Aa) |
| 8 | Marker99922 | 41 | **G** (0.81Aa) | R (0.60Ab) | A (0.55Ab) |
| 9 | Marker5022 | 204 | **C** (0.61Aa) | Y (0.28Bb) |  |
| 10 | Marker46623 | 40 | **A** (0.61Aa) | R (0.34Bb) | G (0.58Aa) |
| 11 | Marker5233 | 18 | **G** (0.62Aa) | R (0.40Bb) |  |

^a^ Numbers in the parentheses are the average MFVWs of the corresponding allele, and different uppercase and lowercase letters represent significance at *P* < 0.01 and *P* < 0.05, respectively; and alleles in bold are favorable.
