# Supplementary figures and images for: Genome-wide association study identifies favorable SNP alleles and candidate genes for waterlogging tolerance in chrysanthemums
Source: Hortic Res. 2019 Feb 1;6:21. doi: 10.1038/s41438-018-0101-7 (PMC6355785; doi:10.1038/s41438-018-0101-7)

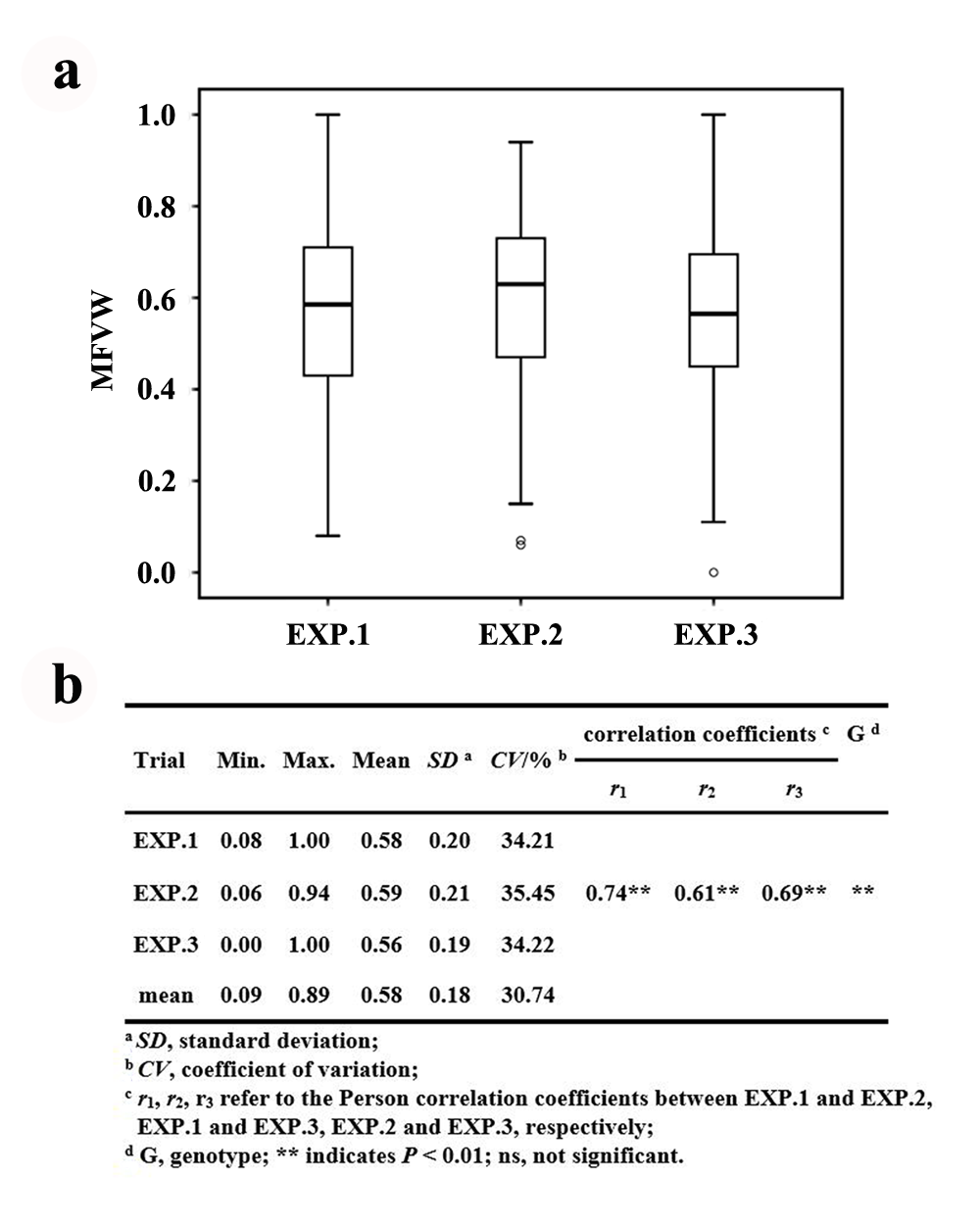

Supplement: Supplementary file 8 — Figure S1 [file 41438_2018_101_MOESM8_ESM.tif]

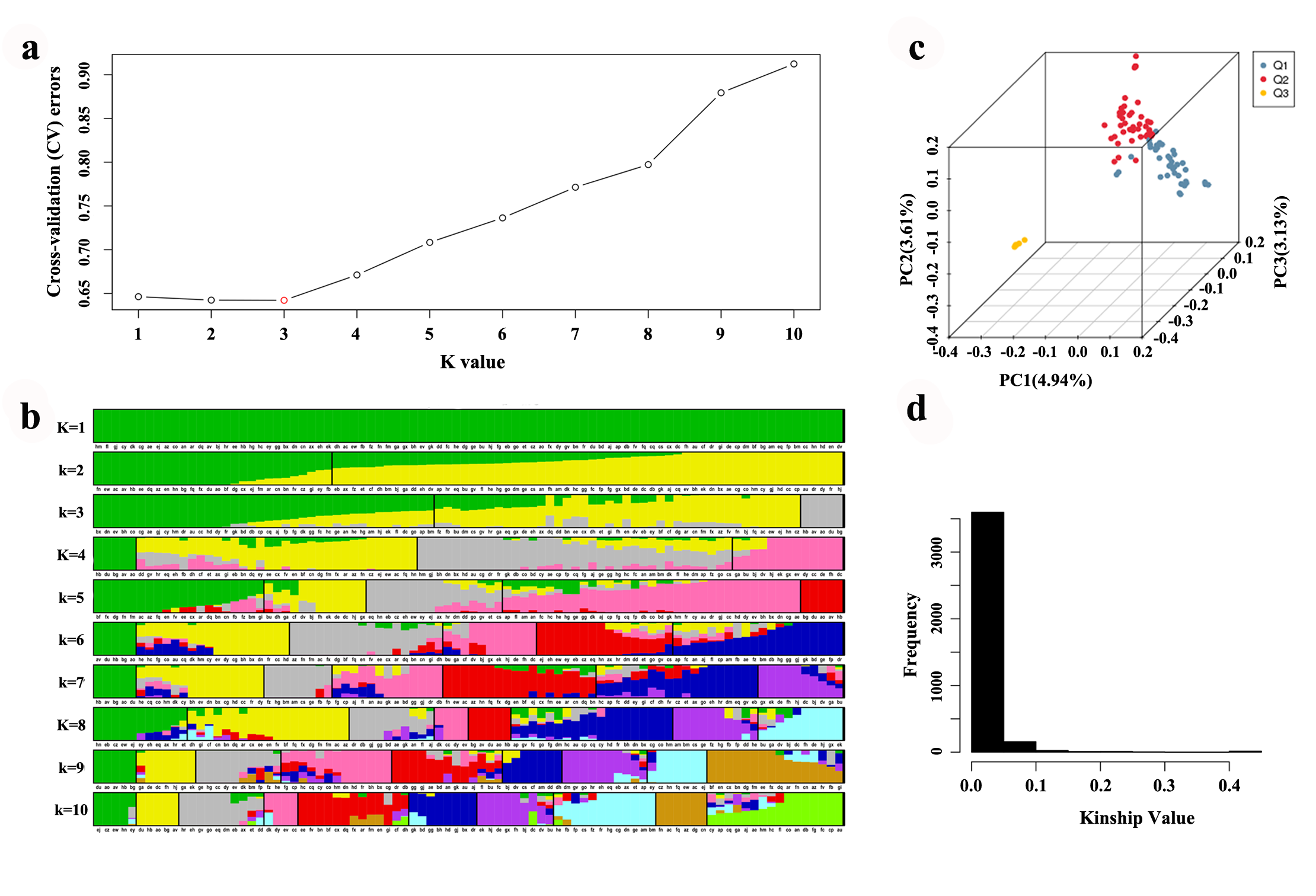

Supplement: Supplementary file 9 — Figure S2 [file 41438_2018_101_MOESM9_ESM.tif]

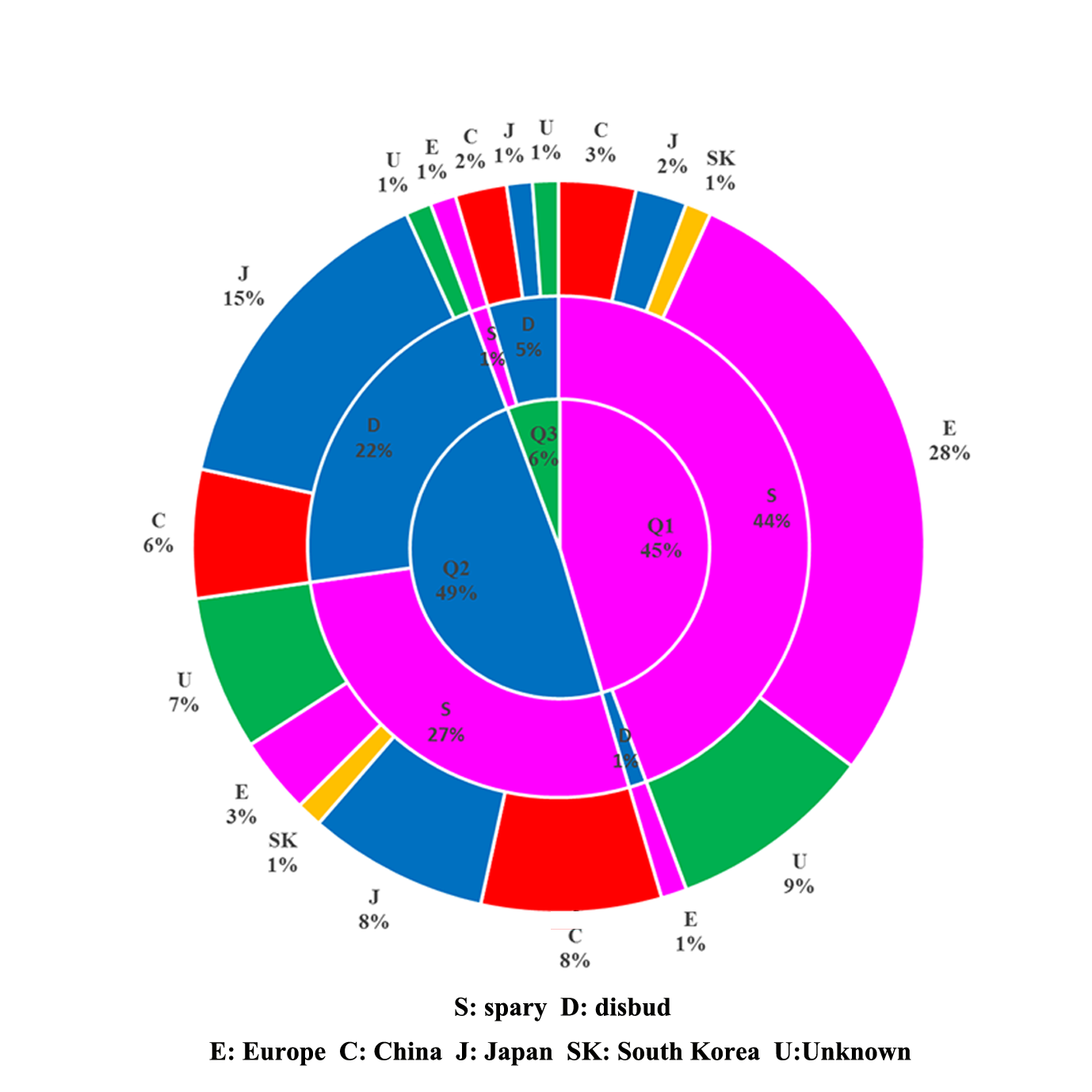

Supplement: Supplementary file 10 — Figure S3 [file 41438_2018_101_MOESM10_ESM.tif]

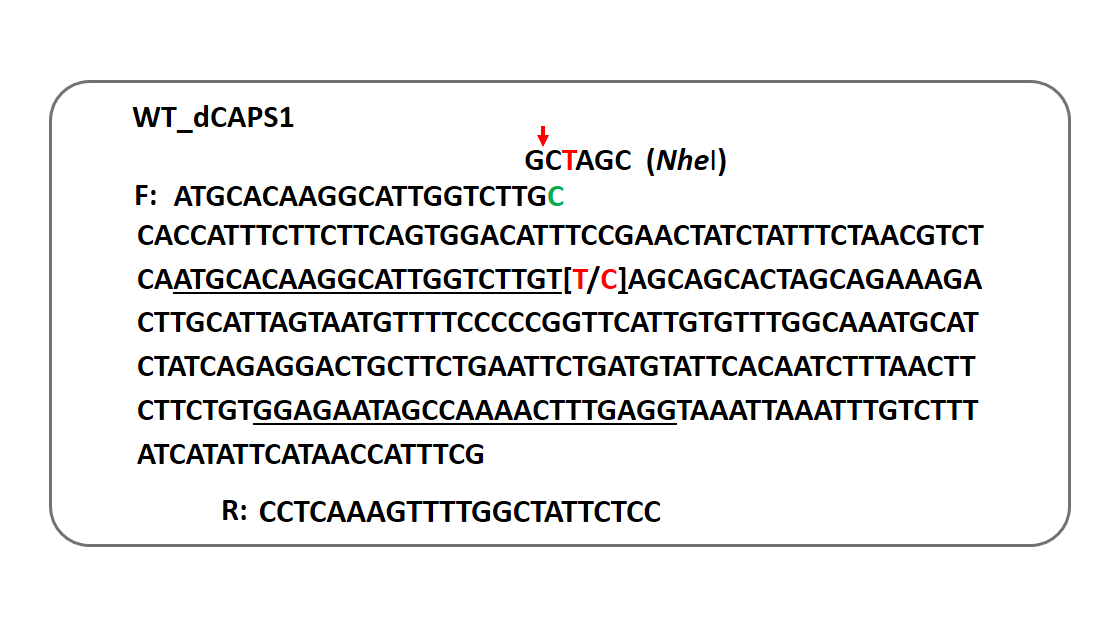

Supplement: Supplementary file 11 — Figure S4 [file 41438_2018_101_MOESM11_ESM.tif]

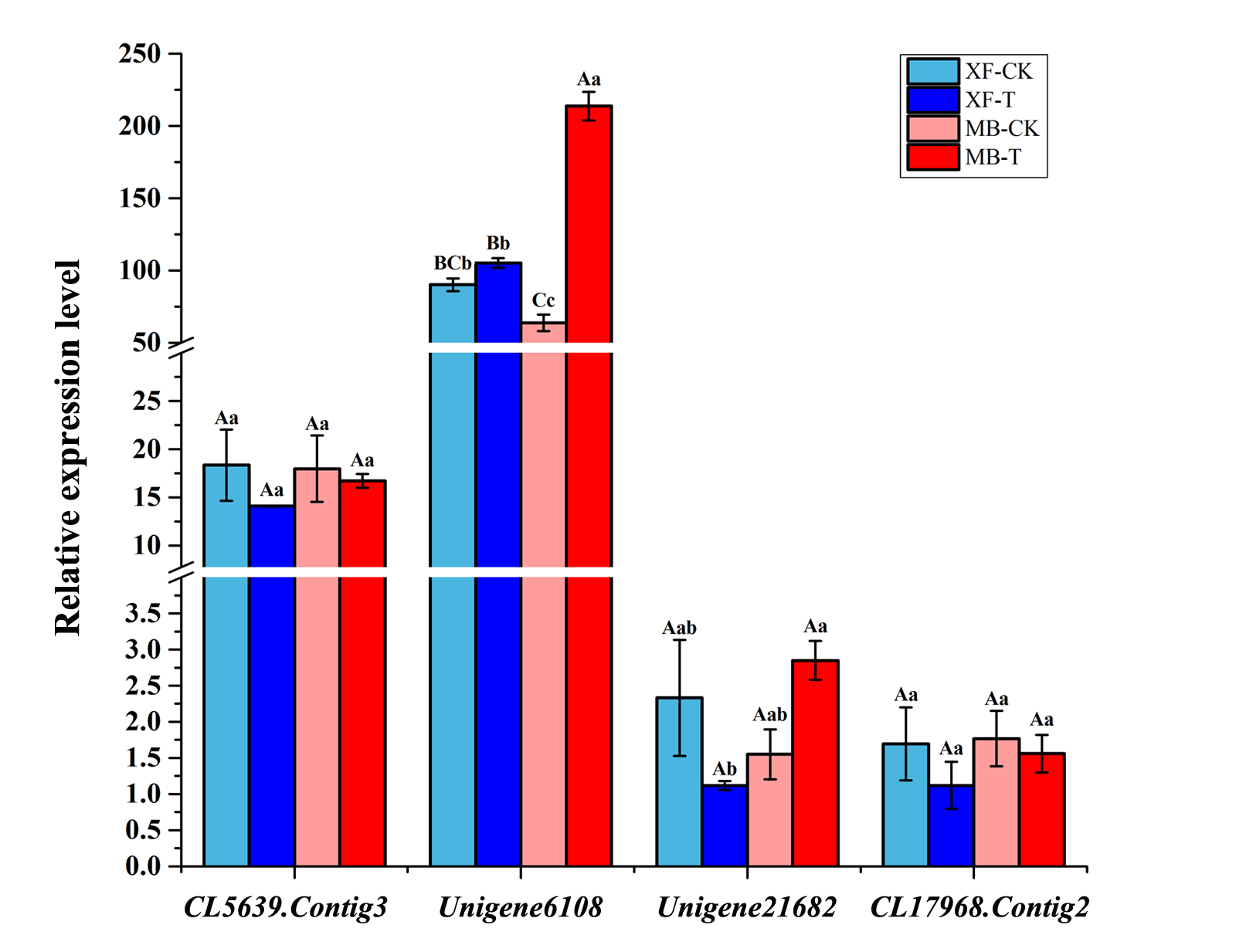

Supplement: Supplementary file 12 — Figure S5 [file 41438_2018_101_MOESM12_ESM.tif]
